# Endothelial Immunity Trained by Coronavirus Infections, DAMP Stimulations and Regulated by Anti-Oxidant NRF2 May Contribute to Inflammations, Myelopoiesis, COVID-19 Cytokine Storms and Thromboembolism

**DOI:** 10.3389/fimmu.2021.653110

**Published:** 2021-06-25

**Authors:** Ying Shao, Jason Saredy, Keman Xu, Yu Sun, Fatma Saaoud, Charles Drummer, Yifan Lu, Jin J. Luo, Jahaira Lopez-Pastrana, Eric T. Choi, Xiaohua Jiang, Hong Wang, Xiaofeng Yang

**Affiliations:** ^1^ Centers of Cardiovascular Research, Inflammation, Translational & Clinical Lung Research, Temple University Lewis Katz School of Medicine, Philadelphia, PA, United States; ^2^ Metabolic Disease Research, Thrombosis Research, Departments of Pharmacology, Microbiology and Immunology, Temple University Lewis Katz School of Medicine, Philadelphia, PA, United States; ^3^ Neurology, Temple University Lewis Katz School of Medicine, Philadelphia, PA, United States; ^4^ Psychiatry and Behavioral Science, Temple University Lewis Katz School of Medicine, Philadelphia, PA, United States; ^5^ Surgery, Temple University Lewis Katz School of Medicine, Philadelphia, PA, United States

**Keywords:** endothelial cell, trained immunity, coronavirus infection, DAMP, oxidative phosphorylation

## Abstract

To characterize transcriptomic changes in endothelial cells (ECs) infected by coronaviruses, and stimulated by DAMPs, the expressions of 1311 innate immune regulatomic genes (IGs) were examined in 28 EC microarray datasets with 7 monocyte datasets as controls. We made the following findings: The majority of IGs are upregulated in the first 12 hours post-infection (PI), and maintained until 48 hours PI in human microvascular EC infected by middle east respiratory syndrome-coronavirus (MERS-CoV) (an EC model for COVID-19). The expressions of IGs are modulated in 21 human EC transcriptomic datasets by various PAMPs/DAMPs, including LPS, LPC, shear stress, hyperlipidemia and oxLDL. Upregulation of many IGs such as nucleic acid sensors are shared between ECs infected by MERS-CoV and those stimulated by PAMPs and DAMPs. Human heart EC and mouse aortic EC express all four types of coronavirus receptors such as ANPEP, CEACAM1, ACE2, DPP4 and virus entry facilitator TMPRSS2 (heart EC); most of coronavirus replication-transcription protein complexes are expressed in HMEC, which contribute to viremia, thromboembolism, and cardiovascular comorbidities of COVID-19. ECs have novel trained immunity (TI), in which subsequent inflammation is enhanced. Upregulated proinflammatory cytokines such as TNFα, IL6, CSF1 and CSF3 and TI marker IL-32 as well as TI metabolic enzymes and epigenetic enzymes indicate TI function in HMEC infected by MERS-CoV, which may drive cytokine storms. Upregulated CSF1 and CSF3 demonstrate a novel function of ECs in promoting myelopoiesis. Mechanistically, the ER stress and ROS, together with decreased mitochondrial OXPHOS complexes, facilitate a proinflammatory response and TI. Additionally, an increase of the regulators of mitotic catastrophe cell death, apoptosis, ferroptosis, inflammasomes-driven pyroptosis in ECs infected with MERS-CoV and the upregulation of pro-thrombogenic factors increase thromboembolism potential. Finally, NRF2-suppressed ROS regulate innate immune responses, TI, thrombosis, EC inflammation and death. These transcriptomic results provide novel insights on the roles of ECs in coronavirus infections such as COVID-19, cardiovascular diseases (CVD), inflammation, transplantation, autoimmune disease and cancers.

## Introduction

The endothelium is a highly specialized, dynamic, disseminated organ with many essential functions in physiological processes (1). More than 60 trillion endothelial cells (EC) constitute the largest interconnected organ in the human body, weighing three kilograms (2). Due to this vast coverage and the nature of being the first cell type to encounter any pathogens in the blood circulation, the endothelium (3) could function as the primary intravascular sentinel system (4–6). Thus, based on the comparison of EC to prototypic innate immune cells such as macrophages (7) in 13 innate immune features, we proposed a new paradigm that EC are innate immune cells (1, 8), which: *1)* system; *2)* acquire function as antigen-presenting cells; *3)* play immune enhancing and immune suppressive roles depending on their cytokine secreting panel; *4)* have plasticity to switch into other cell types (1, 8). EC express major histocompatibility class I (MHC I) molecules for antigen presentation and PAMPs receptors (pattern-recognition receptors, PPRs)/DAMPs (9), which detect PAMPs in their quiescent state (10, 11). A great deal of DNA microarrays have documented changes in gene expression of EC in PAMP- and DAMP- induced inflammatory responses, which have revealed the enrollment of EC in sensing and immediate response to danger signaling at the frontier line to limit tissue damage (12). For example, Toll-like receptors (TLR) recognition in EC of bacterial products results in the secretion of cytokines that recruit other cells (13) and directly activating innate effectors (14). By using tissue-specific TLR pathway component myeloid differentiation primary response 88 (Myd88)-deficient mice, a recent study revealed that EC, not hematopoietic cells, hepatocytes, pericytes, or bone marrow (BM) stromal cells, are the primary source of granulocyte colony-stimulating factor (G-CSF) production in response to lipopolysaccharide (LPS) stimulation (15) or infection with *Escherichia coli* (*E. coli*), which mediated by the EC-intrinsic TLR4/Myd88 signaling pathway (16). This pathogen dissemination process is required to initiate emergency granulopoiesis, skewing myeloid progenitor lineage toward granulocyte-macrophage progenitors then accelerating BM neutrophil generation (17). In addition, by taking advantage of mice that had TLR4 exclusively on the endothelium, the residential sensitivity of anti-pathogen defense was identified in EC and other tissues (4). When exposed to a systemic LPS or intraperitoneal *E. coli* challenge, in contrast to BM-derived immune cells, which were critical for pathogen detection at barrier sites, such as lung, EC mobilize neutrophils to primary sites of infection, clear bacteria, and resist a lethal dose of *E. coli*.

In addition to PAMPs derived from microbes, EC express caspase-1/inflammasomes (18), one type of PAMPs/DAMPs receptors (PRRs) (11), and sense CVD risk factors such as hyperlipidemia (13, 19), hyperhomocysteinemia (20), uremia toxins associated with chronic kidney disease (21), and hypoxia (22), which activate EC and accelerate vascular inflammation (21, 23) and atherosclerosis (24, 25). Mitochondrial ROS (mtROS) and proton leak mediate a newly-termed physiological activation and pathological activation (26–29). Moreover, under the stimulation by proatherogenic lipids chronic disease conditions such as hyperlipidemia, EC have a novel prolonged activation status, as we reported, with four innate immune features including upregulation of EC adhesion molecules (30) and secretion of cytokines and chemokines (31), upregulation of additional DAMP receptors such as CD36, and increased expression of co-signaling receptors and MHC class II molecules (32). Furthermore, acetylation of histone 3 lysine 14 (H3K14) in genomic regions that encode trained immunity enzymes in lysophosphatidylcholine (LPC)-activated human aortic EC (HAEC) are increased in comparison to the genomic areas that encode for EC activation genes (33). These findings suggest that the acetylation of H3K14 participates in mediating innate immune memory (trained immunity, TI) function of EC, which are not suppressed by anti-inflammatory and anti-EC activation cytokines interleukin-35 (IL-35) (15, 34, 35) and IL-10 (34, 36). In addition to facilitating inflammatory cell trans-EC migration and immune responses, EC increase the expression of T cell co-stimulation receptors and immune checkpoint receptors/T cell co-inhibition receptors when stimulated by tumor necrosis factor-α (TNF-α) and interferon-γ (IFN-γ) (37), suggesting that EC may also play immune tolerogenic function during inflammation via reverse signaling of immune checkpoint receptors (7, 38). Similarly, EC execute innate antiviral machinery by inducing expression of several immunomodulatory genes in response to IFNs and to double-stranded RNA (dsRNA) (39); and the early intervention of EC to produce dsRNA protein kinase and activate intrinsic and extrinsic apoptotic pathways is of vital importance in restricting viral dissemination and eliminating viral infection (40). Hence, EC contribute to the early detection and destruction of transformed host cells (1). Once the innate immune responses are disrupted in EC, such as heavily infected with Ebola virus, the immune systems are consequently unable to receive signals to generate a timely inflammatory response; and the infection is usually fatal (41, 42). A comprehensive list of innate immune regulators has been identified and collected in the innate immune database (https://www.innatedb.com/) (43). However, an important question remained whether EC have innate immune transcriptome and trained immunity that are similar to that identified in monocytes and macrophages (8, 44), making cytokine response amplified into cytokine storms.

The spread of severe acute respiratory syndrome coronavirus 2 [SARS-CoV-2, COVID-19 (45)] has already taken on pandemic proportions, affecting over 100 countries in a matter of weeks (46) (also see https://www.nih.gov/health-information/coronavirus). In addition, SARS-CoV and Middle East respiratory syndrome coronavirus (MERS-CoV) are two highly transmissible and pathogenic viruses that emerged in humans at the beginning of the 21^st^ century (47). It has been reported that SARS-CoV-2 invades human cells via receptor angiotensin-converting enzyme II (ACE2) (48). Given the respiratory nature SARS-CoV-2 pathology and ACE2 binding, the lung ACE2-rich epithelium may be the main target during infection (49–51). However, some patients also exhibit non-respiratory symptoms, such as kidney failure, implying that SARS-CoV-2 could also invade other organs (52). SARS-CoV similarly binds to ACE2 (47); and MERS-CoV binds to two host cell surface molecules dipeptidyl peptidase-4 [DPP4, CD26, MERS-CoV receptor (47)] and α2,3-sialic acids (53). Human and primate brain microvascular EC can be infected by coronavirus, which modulate the expression of intercellular adhesion molecule 1 (ICAM-1) and vascular cell adhesion molecule 1 (VCAM-1) and human leukocyte antigen 1 (HLA-1) (54). Cell tropism for human coronaviruses includes ciliated bronchial cells, both type I and II alveolar cells, and EC, the latter of which express ACE2 and DPP4 (55). Human transmembrane serine proteases such as transmembrane serine protease 2 (TMPRSSII) and human airway trypsin-like protease (HAT) cleave and activate human coronavirus strain 229E (HCoV-229E), SARS- and MERS-CoV spike (S) proteins during viral entry (55).

It has been reported that endoplasmic reticulum (ER) stress and oxysterols trigger EC apoptosis, apoptotic ECs and EC denudation may constitute a critical step in the transition to atherosclerotic plaque erosion and vessel thrombosis (56) related to COVID-19 (57). Besides, a study identified that a dual mode of cell death pathways, including apoptosis and necroptosis might lead to the lung damage in the COVID-19 patients (58). Previous reports from our group (59, 60) and others revealed that the virus-triggered cell death pathways might pose anti-viral responses or immune pathogenesis depending on the activation status. We previously reported that SARS-CoV Envelope protein induces T cell death, which may be a molecular mechanism underlying SARS-CoV-induced lymphopenia (59, 60) Post-mortem histology results from COVID-19 patient revealed lymphocytic endothelitis in lung, heart, kidney, liver and endothelialitis of the submucosal vessels, facilitating the induction of cell death (61).

Regardless of the significant progress in understanding EC role in innate immunity, a few important questions remained. *First*, whether the expressions of innate immune regulators in EC are differentially modulated in response to stimulations of PAMPs and DAMPs; *second*, whether EC are equipped with ACE2 to serve as cellular targets for infections by SARS-CoV-2 and other viruses; *third*, whether EC have trained immunity function which amplifies cytokine responses into cytokine storms; and *fourth*, EC undergo various types of cell death in response to virus infections and DAMP stimuli, which trigger thromboembolism and cardiovascular complications of COVID-19. Our new transcriptomic results in addressing these issues provide novel insights on the roles of ECs in coronavirus infections such as COVID-19, CVD, inflammation, transplantation, autoimmune disease and cancers.

## Methods

### EC Transcriptomics Data and Database Content

**Figure 1:**
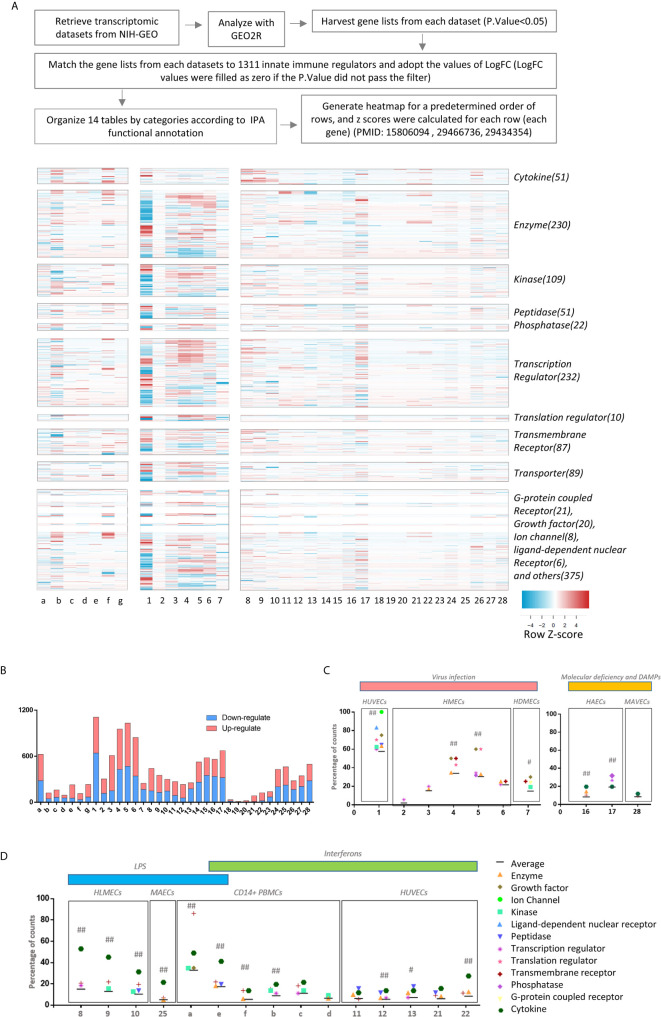
The heat map analyses indicate that 1311 innate immune regulatomic genes are differentially expressed in various endothelial cells. All the microarray datasets were collected from NIH-NCBI Geo DataSets database (https://www.ncbi.nlm.nih.gov/gds/), whose datasets IDs were included. **(A)** Twenty-eight endothelial cell microarray datasets (1-28) were collected from NIH-Geo database in 16 published studies. Twenty-four human and four mouse endothelial cells were included in *in vitro* and *ex vivo* experiments. Specifically, seven comparisons were set in virus infections, four comparisons were set in LPS stimulation, five were set in interferons treatment, nine were set in sterile danger signals, and other situations were associated with key molecules deficiency of innate immune system. Seven datasets of the microarrays (a-g) from human CD14+ peripheral blood mononuclear cells (PBMCs) or CD14+ PBMCs-derived macrophages stimulated with interferon-g (IFNg) or lipopolysaccharide (LPS), which served as the prototypic innate immune cell controls. The datasets were analyzed to maximally mimic the formats used in the original published studies. The expression profiles of 1311 innate immune regulators from a comprehensive innate immune database (https://www.innatedb.com/) in these 35 microarray datasets were analyzed in a panoramic manner. To avoid the bias resulting from the differences between each independent experimental design and techniques, the values of Log fold change (logFC) were adopted in each specific experimental comparison to generate heat map. In addition, cluster analysis grouped the genes that share a similar expression pattern among all samples, regardless of how the data were generated for. A cluster of 1311 genes that shows change (P. value<0.05) in at least one dataset were shown. A color scale was designated the expression signature, where the intensity of the cell's color reflects the value of logFC. Red bar represented the upregulation of gene while blue represented the downregulation of gene (See **Supplementary Table 1**). **(B)** Bar charts displayed the counts of up- and down-regulate genes with P.value<0.05 and |LogFC|>1 in each datasets (See **Supplementary Table 2**). **(C, D)** According to function annotation of Ingenuity Pathway Analysis (IPA), genes with significant change (P.value<0.05 and |LogFC|>1) and all 1311 innate immune genes are categorized, respectively. The category that has a higher percentage than average (Average=counts of gene with significance/1311) in each datasets are displayed in the figure. In order to evaluate the counts of changed gene have statistically significant association with categories, we performed Chi-square tests in JMP pro. ## means P.value (under Prob>ChiSq) <0.001; # means P.value <0.05; (See **Supplementary Table 3**).

EC transcriptomics datasets available in the public domain were collected and organized. To do this, a two-step approach (**Figure 1** and **Supplementary Table S1**) was used: 1) the key words “endothelial OR endothelium” and “Virus”, “endothelial OR endothelium” and “LPS, “endothelial OR endothelium” and “IFNs”) were used to search ArrayExpress EC transcriptomics datasets, and then the title, and abstract of the datasets were screened; and necessary sample information was used to determine which of these studies were performed for gene expression profiling in ECs specifically. Due to intrinsic technical limitation of the datasets, RNA-seq datasets and the studies that data was not made available in the public domain were excluded. Thus, a total of nine human and one mouse studies comprising 26 bulk transcriptomics datasets were analyzed. In addition, we included two endothelial datasets from our previous reports; 2) Four studies comprising seven datasets filtered by key words (“macrophage” and “LPS OR IFNs”) were used as classical innate immune cell controls.

### Transcriptomic Analyses of Innate Immune Regulators in Endothelial Cells

Expression Profiles of 1311 innate immune regulators (IGs) from a comprehensive innate immune database (https://www.innatedb.com/) (43) were collected from 16 published studies deposited in NIH-NCBI Geo DataSets database (https://www.ncbi.nlm.nih.gov/gds/) as we reported (62–64), whose datasets IDs are included in **Table 1**. These datasets included 24 human and four mouse EC from *in vitro* and *ex vivo* experiments. Specifically, seven comparisons were set in virus infection such as influenza virus, MERS-coronavirus, four comparisons were set in LPS stimulation, five were set in interferons treatment, nine were set in danger signals including oxidized phospholipid 1-palmitoyl-2-arachidonoyl-*sn*-glycero-3-phosphorylcholine (oxPAPC) (65), LPC (66), bacterium Borrelia burgdorferi infection (lyme disease agent, https://www.cdc.gov/lyme/index.html), and other situations were associated with key molecule deficiencies of innate immune system. Besides, another seven studies of prototypic innate immune cells such as dendritic cells and macrophages in response to similar stimulations were included as classical innate immune cell controls (**Table 1**). The datasets analyses were processed to approximate the format that was used in the original published studies. To avoid bias resulting from the differences between each independent experimental design and technology, we adopt the value of Log fold changes (logFC) in each specific experimental comparison to generate a heat map (12, 67).

**Table 1:** The information are presented on the profiles of the 35 transcriptomic datasets deposited in NIH-NCBI-Geo Datasets database and EMBL-EBI-ArrayExpress database including 28 datasets collected from twenty-four human and four mouse endothelial cells in *in vitro* and *ex vivo* experiments, and seven datasets collected from dendritic cells and macrophages as controls.

Conditions	Cell origin	Specific comparisons	GEO profiles	Assigned labels
LPS	CD14+ cells from PBMC	LPS stimulation for 24hrs	GSE22886	a
IFN-g		IFN-g stimulation, IFN-g primed + stimulation, and IFN-g primed only for 24 hrs vs control	GSE1925	b,c,d
IFN-g+LPS	CD14+ cells from PBMC	IFN-g + LPS and IFN-g stimulation for 24 hrs	GSE85346	e,f
ZHX2 KO	RAW264.7 Macrophage	Zhx2 null vs WT	PMID 30026271	g
Virus	human umbilical vein endothelial cells (HUVECs)	influenza virus infection vs inoculation of inactivated virus	GSE59226	1
	human microvascular endothelial cells (HMECs)	wild type MERS-CoV(icMERS) infection vs mock for 0hr, 8hrs, 24hrs, 36hrs, 48hrs	GSE79218	2,3,4,5,6
	human dermal endothelial cells (HDMECs)	isolated Kaposi's sarcoma associated herpes virus (KSHV) infection for 7 days vs uninfected	GSE1377	7
LPS	human lung microvascular endothelial cells (HLMECs)	lipopolysaccharide (LPS) exposure for 4hrs, 8hrs, 24hrs	GSE5883	8,9,10
Interferons	HUVECs	IFN-a, IFN-g, IFN-b exposure for 5 hrs	GSE3920	11,12,13
	HUVECs	Notch1 siRNA vs scrambled siRNA	GSE85987	14
		Notch1 siRNA + IL-1b for 4 hrsvs scrambled siRNA + IL-1b		15
	human aortic endothelial cells (HAECs)	oxPAPC treatment for 6 hrs	GSE72633	16
Key molecules deficiency and sterile danger signals exposuer	HACEs	LPC teratment for 18 hrs	PMID 39769317	18
		LPC + IL-35 teratmentvs		19
		LPC for 18 hrs		
		Borrelia burgdorferi infection vs unstimulation		20
	HUVECs	INF-g stimulation vs unstimulation	GSE6092	21
		Borrelia burgdorferi + INF-g vs unstimulation		22
	Fibrosa human aortic valvular endothelial cells (HAVECs)	oscillatory shear exposure for 24 hr vs laminar shear exposture	GSE26953	23
	Ventricularis HAVECs			24
LPS	mouse aortic endothelial cells (MAECs)	LPS exposure for 4 hrs	GSE39264	25
ApoE KO	MAECs	From ApoE-/-mice at four weeks old vs WT mice		26
oxLDL and Oxpapc		oxLDL, oxPAPC exposure for 4 hrs		27,28

### Cluster Analyses of Innate Immune Regulators

As reported (68), cluster analysis groups the genes that shared a similar expression pattern among all samples, regardless of how the data were generated for. A cluster of 1311 genes that showed changes (*p* <0.05) in at least one dataset were shown in **Figure 1A**. A color scale was designated for the expression where the intensity of the cell's color reflected the value of logFC. The red bar represented the upregulation of genes while the blue bar represented the downregulation of genes. Genes are categorized to thirteen categories according to the functional annotations obtained from Ingenuity Pathway Analysis (IPA) as we reported (63).

### Ingenuity Pathway Analysis

IPA using genes with P value <0.05 as expression value cutoff. Gene set enrichment analysis were performed by using Hallmark gene sets from Molecular Signatures Database (MSigDB) (69, 70).

## Results

### The Majority of Modulated Innate Immune Regulators (IGs) Were Upregulated in the First 12 Hours Post-Infection and Maintained Until 48 hr PI in Human Microvascular Endothelial Cells Infected by Middle East Respiratory Syndrome-Coronavirus (MERS-CoV)

Based on progress in the field and our own reports, we proposed a new working model that EC are innate immune cells (1, 8). However, a panoramic view on transcriptomic changes of whole IGs in EC remained unknown. We hypothesized that EC have transcriptomic changes in response to PAMPs from viruses, bacterial components and DAMPs derived from the risk factors of cardiovascular and metabolic diseases such as hyperlipidemia, and disturbed shear stress. To test this hypothesis, we filtered around 1000 transcriptional-profiling experimental datasets of EC deposited in the European Molecular Biology Laboratory-European Bioinformatics Institute (EMBL-EBI) ArrayExpress repository (https://www.ebi.ac.uk/arrayexpress/) and the NIH-Geo database (https://www.ncbi.nlm.nih.gov/gds/) and selected 28 EC microarray datasets (**Table 1**). We argued that transcriptional profiling would provide a wealth of information about many specific innate immune reactions, and the systematic comparison of multiple different datasets would identify common and specific gene expression patterns that might provide further insights into the innate immune response applied by EC. The results show that: *1)* influenza virus (RNA virus) infection in human umbilical vein EC (HUVEC) (GSE59226) led to significant IG expression changes with 468 out of 1311 (35.7%) gene upregulation and 641 out of 1311 (48.9%) gene downregulation (**Figure 1B** and **Supplementary Table 2**); *2)* a special group of IGs were upregulated and downregulated in human microvascular EC (HMECs) stimulated with wild-type middle east respiratory syndrome coronavirus (MERS-CoV, RNA virus) (icMERS) and collected at five time points 0, 12, 24, 36, 48 hours (hr) post-infection (PI) (GSE79218), with upregulated 188 IGs (14.3%)/downregulated 117 IGs (8.9%) at 0 h PI, upregulated 460 IGs (35.1%)/downregulated 150 IGs (11.4%) at 12 hr PI, upregulated 526 IGs (40.1%)/downregulated 428 IGs (32.6%) at 24 hr PI, upregulated 563 IGs (42.9%)/downregulated 469 IGs (35.8%) at 36 hr PI, upregulated 502 IGs (38.3%)/downregulated 342 IGs (26.1%) at 48 hr PI, respectively. Of note, the majority of MERS-CoV-modulated IGs were upregulated rather than downregulated at 12 hr PI, which lasted more than 36 hr PI and started to decrease at 48 hr PI. Since the numbers of IGs upregulated were three times higher in 24 hr PI than 0 hr, suggesting that trained immunity (8, 33, 44) with significantly enhanced response as a novel mechanism may play a significant role in ECs in enhancing innate immune response to MERS-CoV infection; and *3)* Kaposi's sarcoma-associated herpesvirus (KSHV, double-stranded DNA virus) infection, which primarily brings down the cellular innate immune response in B cells (71), downregulated 12.7% GIs in endothelial cells (primary human dermal EC, HDMEC), more than twice as the upregulated ones (6.2%) (72).

Taken together, these new results showed that *first*, human ECs, regardless of the tissue origins of human ECs (umbilical vein, microvascular, or dermal), can be infected by various RNA viruses and DNA viruses, suggesting that human EC express influenza virus receptor α2,6- or α2,3-linked sialic acid (73), MERS receptor dipeptidyl peptidase-4 (DPP4, CD26) (74), and KSHV receptor heparin sulfate proteoglycans containing proteins (75); *second*, the innate immune regulators were significantly upregulated in the first 12 hr PI and maintained until 48 hr PI in HMEC infection by MERS-CoV, suggesting that EC activation at the early stage of viral infection may contribute to inflammatory cell migration from circulating blood to infected tissues and vessels; *third*, since MERS-CoV belongs to the coronavirus family, the same as severe acute respiratory syndrome coronavirus 2 (SARS-CoV2), SARS-CoV2 might also infect the ECs, in which the SARS-CoV2 receptor ACE2 is expressed; and *fourth*, the IGs upregulated by influenza virus infection in HUVECs are significantly different from those upregulated by MERS-CoV in HMECs, and those upregulated by KSHV in HDECs. During KSHV infection of EC, IFN-γ-inducible protein 16 (IFI16) (76) interacts with the adaptor molecule Apoptosis-associated speck-like protein containing a CARD (ASC) and procaspase-1 to form a functional protein complex inflammasome initially detected in the nucleus and subsequently in the perinuclear area (25). KSHV gene expression and/or latent KSHV genome is required for inflammasome activation; and IFI16 colocalizes with the KSHV genome (77, 78) and Epstein-Barr (EB) virus (79) in the infected cell nucleus. In contrast, influenza virus (80) and MERS-CoV-homologous SARS-CoV (81) activates inflammasomes from the cytosol (5, 82). Future studies are needed to determine whether those differences result from tissue origins of ECs, viral replications in cytosol or nucleus, or the types of viruses. These results have demonstrated that EC, as innate immune cels (1, 8), can be infected by viruses, which may contribute to the pathogenesis of viral infection-induced vascular inflammation, facilitation of inflammatory cell migration to infected tissues and systemic infections, disseminated intravascular coagulopathy and thrombosis (83).

### The Expressions of Innate Immune Regulators Were Significantly Modulated in 21 Transcriptomic Datasets From Human ECs Stimulated by Various PAMPs/DAMPs Including LPS, IFNs, Notch 1 siRNAs, oxPAPC, LPC, Shear Stress, Hyperlipidemia and oxLDL 

It has been well accepted that EC play significant roles in facilitating inflammatory cell recruitment into arteries and accelerating atherosclerosis (3, 5, 11). Recently, others and we reported that EC are capable in recognizing DAMPs and conditional DAMPs (9, 66) derived from metabolic diseases such as hyperlipidemia. However, how those DAMPs and conditional DAMPs modulate the expressions of IGs in ECs have not been compared. We hypothesized that the expressions of IGs in ECs are modulated in ECs by various DAMPs and conditional DAMPs (9). To examine this hypothesis, we collected 21 transcriptomic datasets from EC stimulated by various PAMPs/DAMPs and conditional DAMPs including lipopolysaccharide (LPS) (15, 30), interferons (IFNs), proinflammatory master regulator Notch 1 siRNAs, TLR4 antagonist oxidized 1-palmitoyl-2-arachidonyl-sn-glycero-3-phosphorylcholine (PAPC) (oxPAPC) (65) [most of the truncated oxidized phospholipids induce vascular leak and exacerbate inflammation (84)], proatherogenic lipids lysophosphatidylcholine (LPC) (26, 32, 66, 85, 86), shear stress (19), hyperlipidemia and oxidized low density lipoprotein (oxLDL) (13, 34). The results showed in **Figure 1B** and **Supplementary Table 2** that the expressions of: ***a)*** innate immune regulators were significantly modulated in human lung microvascular ECs stimulated by TLR4 agonist LPS over a time course of 4, 8 and 24 hr after LPS stimulation in comparison to non-stimulated controls. The expressions of 292 out of 1311 (22.3%) IGs were upregulated, and 149 out of 1311 (11.4%) were downregulated at 4 hr; 223 (17%) upregulated/129 (9.8%) downregulated at 8 hr; 153 (11.7%) upregulated/146 (11.1%) downregulated at 24 hr, suggesting that LPS induces innate immune responses in EC at 4 - 8 hr. These results correlated well with our previous report (15); ***b)*** IGs were significantly modulated in HUVECs stimulated by IFNα (180 (13.7%) upregulated/89 (6.8%) downregulated), IFNγ (176 (13.4%) upregulated/56 (4.3%) downregulated), and IFNβ (79 (6.0%) upregulated/176 (13.4%) downregulated) for 5 hr in comparison to non-stimulated controls. These results were well correlated with that we reported for the significant roles of IFNα in anti-tumor immune responses (87–89) and the roles of IFNγ in modulating EC immune responses (37); ***c)*** innate immune regulators were significantly modulated in HUVECs stimulated by Notch 1siRNA with 263 genes (20.1%) upregulated/262 (20.0%) genes downregulated), Notch 1 siRNA plus interleukin-1β (IL-1β) with 231 genes (17.6%) upregulated/350 (26.7%) genes downregulated), and Notch 1siRNA with 224 genes (17.1%) upregulated/336 (25.6%) genes downregulated at 5 hr in comparison to non-stimulated controls; ***d)*** IGs were significantly modulated in HAECs stimulated by TLR4 antagonist oxPAPC with 353 genes (26.9%) upregulated/320 (24.4%) genes downregulated for 6 hr in comparison to non-stimulated controls. It is interesting to see that *first*, anti-inflammatory oxidized phospholipids oxPAPC (90) modulate the expressions of equal numbers of upregulated and downregulated IGs in ECs; and *second*, the total numbers (51.3%) of oxPAPC-modulated IGs were the highest among that in ECs stimulated by conditional DAMPs and DAMPs; ***e)*** IGs in the datasets #18 and #19 were significantly modulated in HAECs stimulated by proatherogenic lipids LPC with 17 genes (1.3%) upregulated/16 genes (1.2%) downregulated for 18 hr in comparison to non-stimulated controls. The expressions of IGs were significantly modulated in HAECs stimulated by proatherogenic lipids LPC plus anti-inflammatory cytokine IL-35 with 5 (0.4%) genes upregulated/6 (0.5%) genes downregulated for 18 hr in comparison to non-stimulated controls. Although these numbers were smaller than other groups above discussed, the results were well correlated with that we reported (26, 32, 34, 36); ***f)*** IGs were significantly modulated in HUVECs stimulated by Lyme disease agent Borrelia burgdorferi with 21 genes (1.6%) upregulated/1 gene (0.08%) downregulated in comparison to non-stimulated controls. In addition, the expressions of IGs were significantly modulated in HUVECs stimulated by proinflammatory/Type 1 T helper cell (Th1) cytokine IFNγ with 75 genes (5.7%) upregulated/10 gene (0.8%) downregulated in comparison to controls. Of note, the numbers of IGs in HUVECs stimulated by IFNγ for 8 hr were different from that in the dataset #12, also with IFNγ stimulation for 5 hr, suggesting the roles of different stimulation time. Moreover, the expressions of IGs were significantly modulated in HUVECs stimulated by Lyme disease agent bacteria Borrelia burgdorferi plus IFNγ with 103 genes (7.9%) upregulated/21 gene (1.6%) downregulated in comparison to controls. The results were correlated well with that reported, IFNγ alters the response of Borrelia burgdorferi-activated endothelium to favor chronic inflammation (91); ***g)*** IGs were significantly modulated in fibrosa human aortic valve ECs (fHAVECs) stimulated by oscillatory shear with 70 genes (5.4%) upregulated/69 genes (5.3%) downregulated in comparison to laminar shear controls. In addition, the expressions of IGs were significantly modulated in ventricularis human aortic valve ECs (vHAVECs) stimulated by oscillatory shear stress with 227 genes (17.3%) upregulated/202 genes (15.4%) downregulated in comparison to laminar shear controls (92); and ***h)*** IGs were significantly modulated in mouse aortic ECs (MAECs) stimulated by LPS for 4 hr with 238 genes (18.2%) upregulated/224 genes (17.1%) downregulated in comparison to controls. In addition, the expressions of IGs were significantly modulated in MAECs from atherogenic apolipoprotein E deficient (ApoE KO) mice with 115 genes (8.8%) upregulated/166 genes (12.7%) downregulated in comparison to that from wild-type controls (93). Moreover, the expressions of IGs were significantly modulated in MAECs stimulated by oxidized oxLDL for 4 hr with 139 genes (10.6%) upregulated/206 genes (15.7%) downregulated in comparison to controls; and MAECs stimulated by oxPAPC for 4 hr with 215 genes (16.4%) upregulated/281 genes (21.4%) downregulated in comparison to controls.

**Figure 2:**
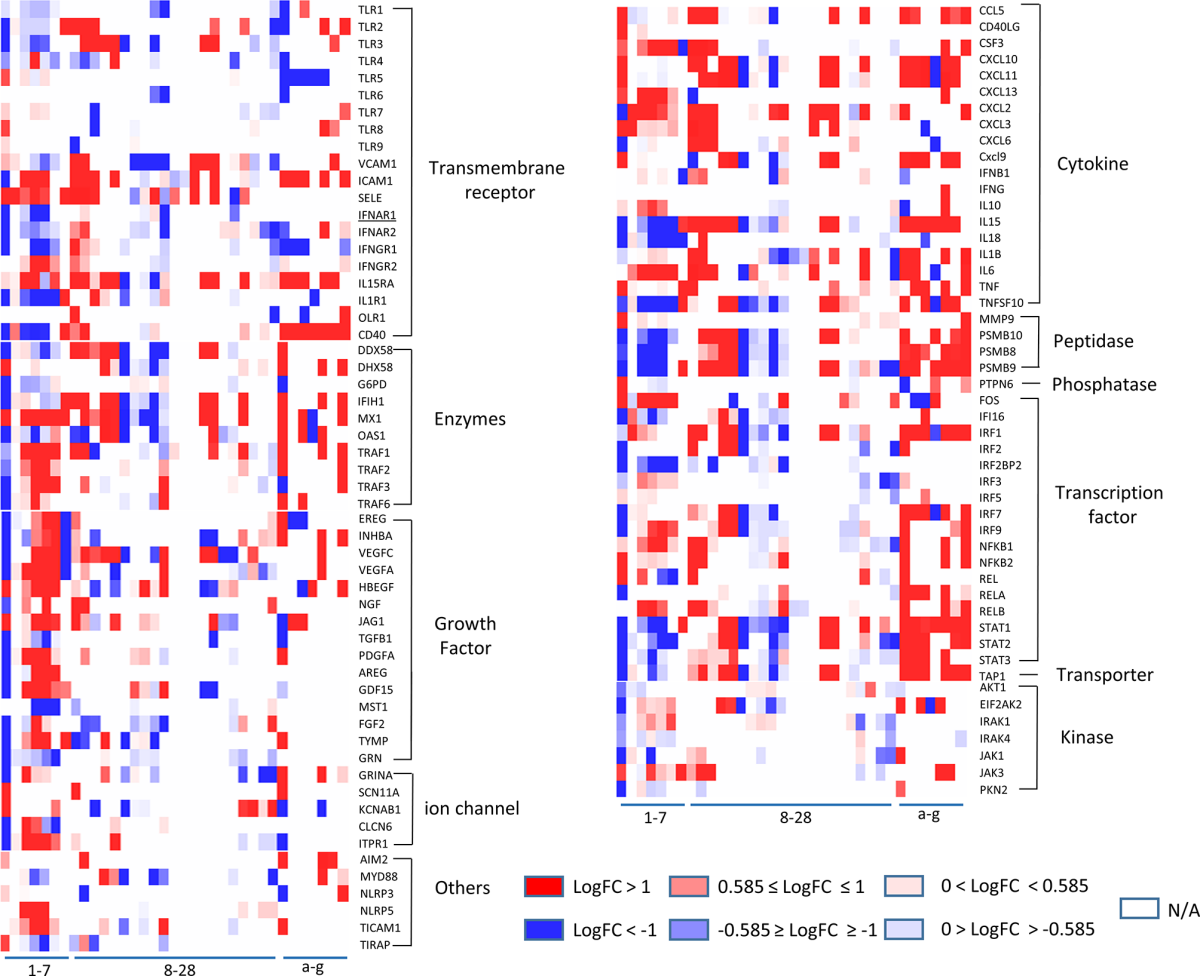
The heatmap plot with customized color scales indicates that some of 11 functional groups of innate immune regulatomic genes are shared in endothelial cells infected by viruses and stimulated by DAMPs. The values of LogFC (P value <0.05) in each comparison are colored via the color scales. The datasets (Cells) in blank (N/A) indicates that differentiation expression (DE) of the gene has no statistical significance (*P* value ≥ 0.05) in the comparisons. The gene categories were identified by the functional annotations in the Ingenuity Pathway Analysis (IPA).

Taken together, our EC transcriptomic data have demonstrated the following: *first*, the differential expressions of IGs in ECs stimulated by various PAMPs/conditional DAMPs are even remarkable than that observed in the transcriptomic data from prototypic innate immune cells such as macrophages with equivalent stimuli (**Figure 1B**). Once again, our results suggest that ECs are innate immune cells not only in virus infections or PAMPs-stimulation but also in CVD risk factor-derived DAMPs and conditional DAMPs (1, 8); *second*, IGs are more upregulated than downregulated (**Figure 1B** and **Supplementary Table 2**) upon multiple stimuli, including early stage of LPS stimulation, IFNα/IFNγ, Borrelia burgdorferi (BB) plus IFNγ, suggesting that upregulated IGs play more important roles in promoting EC activation and other innate and adaptive immune responses; *third*, human ECs present different IGs expression pattern from mice ECs in response to oxPAPC stimulation, which mimics several pro- and anti-inflammatory effects induced by oxidized lipoproteins (94); and *fourth*, proatherogenic stimuli including oscillatory shear stress, oxLDL, IFNs, LPS, Notch 1, hyperlipidemia, and LPC significantly upregulate IGs in various ECs with different scales, suggesting that ECs of different origin have different sensitivity in response to proatherogenic stimuli, and those changes of IG in ECs play critical roles in promoting vascular inflammation and atherogenesis.We examined a hypothesis that the expressions of different functional groups of IGs are modulated in response to various pathophysiological stimuli in EC. The gene categories were followed by the functional annotations obtained from Ingenuity Pathway Analysis (IPA). As shown by the heat map in **Figure 2**, IGs were classified into ten major groups, including cytokines (51 IGs), enzymes (230 IGs), kinases (109 IGs), peptidases (51 IGs), phosphatases (22 IGs), transcription factors (232 IGs), translational regulators (10 IGs), transmembrane receptors (87 IGs), transporters (89 IGs), and the last diversified group with G-protein coupled receptors (21 IGs), growth factors (20 IGs), ion channels (8 IGs), ligation-dependent nuclear receptors (6 IGs), and other regulators (375 IGs). In order to evaluate the changed genes with a statistically significant association in each category, we performed Chi-square tests. The results in **Figures 1C, D** showed that the counts of changed genes have statistically significant association with categories in 16 datasets: *1)* In influenza virus infected ECs, IGs in multiple categories were above average, especially ion channels and ligation-dependent nuclear receptors; *2)* In MERS-infected ECs, IGs in growth factor and transmembrane receptor have the most impact at 24 hr PI, IGs in growth factor and translation regulator have the most impact at 36 hr PI ; *3)* Upon LPS and IFN-γ treatments, ECs from human and mouse both exhibited that IGs in cytokines have the most impact, similar as CD14^+^ PBMC presented (95); *4)* In IFN-β treated EC, in addition to cytokines, IGs in peptidase also have the most impact on the significant association; and *5)* Although IGs in enzymes and transcription factors were not rank the highest impact, IGs in these two categories devoted to the significance more than average in ECs upon virus infection and IFN-γ stimulation.

Our results taken together have demonstrated that growth factors, enzymes, and transcription factors are among the most dynamic changed IG groups in virus-infected endothelial cells, whereas cytokines are the most significantly upregulated IG group shared by ECs stimulated with various stimuli.

To further zoom in on the significantly upregulated IGs, the expression patterns of 106 out of 1311 IGs (8.1%), including TLRs (11, 96), signaling adaptors and transcription factors, those closely related to classical innate immunity, were selected to be presented in **Figure 2**. The results showed that *i)* LPS induced a strong increase in TLR2 mRNA but not in TLR1, TLR4 and TLR6 mRNA in HLMECs, similar to the change in umbilical vein derived EC, when receiving TNF-α, LPS or IL-1beta administration (97). Meanwhile, the expression level of transcription factors RELB, NFKB1, and NFKB2 increased, which may responsible for the induction of TLR2 by inflammation stimuli. In addition, two RLRs, DDX58 (RIG-I) and IFIH1 (MDA5), were induced by LPS, similar to what had been found in macrophages (98, 99). *ii)* TLR3, a receptor for viral dsRNA, displayed a strong reduction in influenza virus and icMERS infection, while TICAM1, TRAF1, TRAF2, TRAF3 and TRAF6, adaptors responsible for TLR3, changed in an opposite direction with TLR3. Similar expression pattern happened on the IFN regulatory factor IRF3, IRF9, which could induce type I IFN production (100, 101). Although no impressive change of IFNG, IFNB shown in the endothelial cells in current collected data, IFNGR1, IFNAR1, and IFNAR2, the receptors of IFN- γ and IFN-α exhibited similar change as TLR3; *iii)* Different from IFN-α and IFN-β, IFN-γ treatment uniquely induced TLR4 expression in HUVECs (102) but caused a less up-regulation of adaptors TRIF (TICAM1) and MYD88; *iv)* Interferon regulatory factor (IRF) family members, such as IRF1, IRF2, IRF7 and IRF9, displayed a strong elevation upon the type I IFN stimulation, similar to the change in macrophage and DCs. While IRF3 (103), one of the most well characterized transcription factors involved in the regulation of innate immune responses, had response to neither type I IFN nor LPS stimulation at the transcription level in human ECs. Of note, IRF3 in MAECs exhibited decreased transcriptional level upon oxLDL and oxPAPC treatments; *v)* STAT1, STAT2, STAT3, members of transcriptional regulator family which modulate the crucial aspect of innate and adaptive immunity 28, exhibited downregulated transcription level in HUVECs with active influenza virus infection, and in HMECs with icMERS as well. Taken together, these results have demonstrated that a large number of innate immune mediators and regulators in ECs presented sensitive reactions to various viruses infection and stimulations by DAMPs; the upregulations of TLR3 (104), AIM2 (105, 106), IFI16 (25), IRF3, IRF5, IRF7, and NF-kB (105) suggest upregulation of sensors for nucleic acid dangers (107, 108) during virus infection, neurodevelopment (106), and vascular diseases (104); and a set of upregulated IGs upregulated in EC are shared among EC infected by viruses and EC stimulated by DAMPs such as nucleic acid sensors, suggesting ECs use similar or same innate immune mechanisms at least partially in virus infections and DAMP stimulations.

### Human Heart EC and Mouse Aortic EC Express All Four Types of Coronavirus Receptors Such as ANPEP, CEACAM1, ACE2, DPP4 and Virus Entry Facilitator TMPRSS2 (Human Heart); Most of Coronavirus Replication-Transcription Protein Complexes Are Expressed in Human Microvascular EC, Which Contribute to Viremia, Thromboembolism, and CVDs 

**Figure 3:**
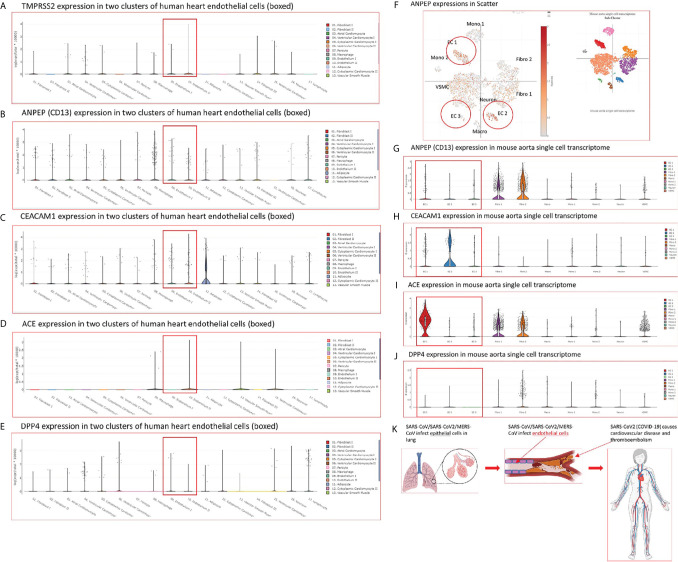
**(A–E)** The mRNA transcripts of four types of coronavirus receptors such as membrane alanyl aminopeptidase (ANPEP, CD13, receptor for human coronavirus-229E), carcinoembryonic antigen family cell adhesion molecule 1 (CEACAM1, receptor for mouse hepatitis virus), angiotensin-converting enzyme 2 (ACE, receptor for SARS-CoV, SARS-CoV2), and dipeptidyl peptidase-4 (DPP4, receptor for MERS-CoV) as well as TMPRSS2 are found in two clusters of human heart endothelial cells. The data mining analyses were performed on the Single Cell RNA-Seq database of the Broad Institute of MIT and Harvard (Single CellBeta Portal; https://singlecell.broadinstitute.org/single_cell/study/SCP498/transcriptional-and-cellular-diversity-of-the-human-heart#study-summary). **(F–J)** The mRNA transcripts of four types of coronavirus receptors such as ANPEP, CEACAM1, ACE, and DPP4 are found in mouse aortic endothelial cell clusters. The data mining analyses were performed on the Single Cell RNA-Seq database of the Broad Institute of MIT and Harvard (Single CellBeta Portal; https://singlecell.broadinstitute.org/single_cell/study/SCP289/single-cell-analysis-of-the-normal-mouse-aorta-reveals-functionally-distinct-endothelial-cell-populations#study-summay, PMID: 31146585). **(F)** ANPEP expressions in three endothelial cell clusters were circled in red in the Scatter; **(G)** ANPEP expressions in three endothelial cell clusters were boxed in red in the Distribution; **(H)** CEACAM1 expressions in three endothelial cell clusters were also boxed in read in the Distribution; **(I)** ACE expressions in three endothelial cell clusters were also boxed in read in the Distribution; **(J)** DPP4 expressions in three endothelial cell clusters were also boxed in read in the Distribution. **(K)** A new working model: Infection of vascular endothelial cells by SARS-CoV2/MERS-CoV causes innate immune responses in endothelial cells, which may induces cytokine storm, and triggers thromboembolism. The part of figure was created with BioRender.com.

SARS-CoV2 virus, the etiological agent of coronavirus disease 2019 (COVID-19), can directly infect immune and EC by docking viral proteins (109). To consolidate this finding, we hypothesized that EC express all the four coronavirus receptors identified, including membrane alanyl aminopeptidase (ANPEP, CD13, a receptor for human coronavirus-229E), carcinoembryonic antigen family cell adhesion molecule 1 (CEACAM1, a receptor for mouse hepatitis virus), angiotensin-converting enzyme 2 (ACE2, a receptor for SARS-CoV, and SARS-CoV2), and dipeptidyl peptidase-4 (DPP4, a receptor for MERS-CoV) as well as transmembrane serine protease 2 (TMPRSS2) (110, 111). To test this hypothesis, we searched the expression of these four receptors and coronavirus S protein priming serine protease TMPRSS2 (112) in human EC in the MIT-Harvard Broad Institute Single Cell^Beta^ Porter single-cell RNA-Seq database, Study: Transcriptional and Cellular Diversity of the Human Heart (https://singlecell.broadinstitute.org/single_cell/study/SCP498). As shown in **Figures 3A–E**, all the four types of coronavirus receptors were expressed in two EC clusters in the human heart. In addition, as shown in **Figures 3F–J**, all the four types of coronavirus receptors were also expressed in three EC clusters in mouse aorta in another single-cell RNA-Seq dataset, Study: Single-cell analysis of the normal mouse aorta reveals functionally distinct EC populations (https://singlecell.broadinstitute.org/single_cell/study/SCP289) (113). Of note, TMPRSS2 expression was not found in mouse aortic EC. In addition to expression evidence of coronavirus entry receptors in EC, we further examined a hypothesis that coronavirus replication-transcription host cell protein complexes (114) (RTC) are expressed in EC and other cells. As shown in **Table 2**, coronavirus replication-transcription host cell protein complexes included 52 host factors based on their significances to virus replication and transcription, including five translation factors, 20 transport factors, six catabolic process factors, two cell organization factors, 19 other factors. We found that in the transcription level, a major part of RTC genes (p<0.05) in ECs exhibited a down-regulation at early time points (12 hr, 24 hr PI), while started to reverse along with the infection time. It suggested that ECs regulated key host responses from anti-virus to a pro-virus replication environment during the MERS infection. To better understand the host response outcomes through RTC genes during the infection timeline, we also compared the transcription level of RTC in icSARS-CoV infected Calu-3 human lung cells. The results showed that Calu-3 cells had an overwhelming number of RTC downregulated from 36 hr to 48 hr, and the down-regulation became diminished at 72 hr. Of note, parts of the downregulated factors were increased when icSARS-CoV was mutated in the open reading frame 6 (ORF6) (115), indicating ORF6 in icSARS-CoV not only attenuated the activity of host transcription factors that are critical for establishing antiviral responses (115), but, to some extent, lessened the host favor of virus replication. In addition, in mouse lung tissue infected by SARS-CoV (116), no significant downregulation RTC genes were found, and the increase of several RTC genes started around 4 to 7 days, suggesting the differences of host reaction between *in vitro* and *in vivo*. Taken together, our results have demonstrated that, *first*, human heart EC and mouse aortic EC express all four types of coronavirus receptors such as ANPEP, CEACAM1, ACE2, DPP4 and virus entry facilitator TMPRSS2 (human heart endothelial cells); *second*, most of coronavirus RTC are expressed in human microvascular EC, which may contribute to coronavirus viremia, thromboembolism, and CVDs (**Figure 3K**); and *third*, use of RTC for coronavirus replication and transcription may have cell-type specificity.

**Table 2:** The expressions of majority 43 out of 52 (82.7%) (non-changed and upregulated) coronavirus replication-transcription complex (RTC)-proximal host factors (PMID: 30632963) in coronavirus-infected cells indicate that endothelial cells and other cells may use these factors for viral replication and RNA transcription,which may be novel therapeutic targets.

		HMECs				Calu-3 cells										Mouse Lung tissues		
Gene symbol	Significance	ic-MERS				ic-SARS					ic-SARS deltaORF6 mutant					MA-15 (SARS-CoV)		
		[with 5 PFU (plaque-forming unit) per cell]				(with 105 PFU per mouse)										(with 105PFU per mouse)		
		12(hr)	24(hr)	36(hr)	48(hr)	12(hr)	24(hr)	36(hr)	48(hr)	72(hr)	12(hr)	24(hr)	36(hr)	48(hr)	72(hr)	2(d)	4(d)	7(d)
*Translation*																		
EIF3E	****							-1.56	-1.70	-1.10								
EIF3F	****		-1.55	-1.49				-2.01	-2.46	-1.45			-1.22	-1.82	-1.57			
EIF3I	****						-1.18	-1.91	-1.83				-1.21	-1.20				
RPL13A	****		-1.25	-1.18				-1.47	-1.48	-1.09					-1.12			
RSL24D1	**							-1.41	-1.65									
*Transport*																		
AAK1	*	1.73	-1.51	-1.43			-1.15							1.35	1.06			
ACBD5	**				-1.09		-2.44	-1.49	-1.46									
BTF3	**	-2.01	-2.05	-1.71				-1.73	-1.98	-1.17			-1.01	-1.29	-1.06			
CLINT1	*			1.00				-1.44	-1.46	-1.18								
DNM1L	*							-1.02										
GBF1	***													1.52				
KIF11	***		-1.19		2.06		-1.03	-1.28	-2.04								2.30	2.25
KPNB1	**							-1.00	-1.09									
NACA	**							-1.76	-1.85	-1.25			-1.07	-1.21	-1.10			
RANGAP1	*		1.16	1.27														
SCFD1	**							-1.09										
SNRPE	****	-1.46					-1.21	-1.92	-1.77				-1.27	-1.34				
SNX9	**							-1.05	-1.15									
SRP54A	***																	
SRP68	*			1.00				-1.18										
STX5A	**																	
TNPO1	*																	
VAPA	*			1.23			-1.02	-1.44	-2.14	-1.42				1.09	1.33			
YKT6	**							-1.42	-1.41									
ZFYVE1	*							-1.40	-1.03					1.73	1.63			
*Catabolic process*																		
DNAJC10	*	-1.10					-1.06	-1.71	-1.84	-1.93			-1.26	-1.77	-1.83			
FAM134B	*							-1.96	-2.93	-2.04			-1.17	-1.69	-1.09			
PSMB3	*						-1.24	-2.10	-1.82				-1.57	-1.53	-1.02			
PSMC2	**							-1.38	-1.09									
PSMD1	***				1.17			-1.10										
TFEB	**	-4.41	-4.73	-4.23	-3.41													
*Cell organization*																		
CKAP5								-1.34	-1.40									
PXN	*		-1.13					-1.56	-1.56	-1.12								
*Others*																		
BAG3	*	1.88	5.25	4.84	2.76									1.82	3.04	2.55		
BPNT1	*	-1.49	1.86	-1.62	-1.07		-1.67	-1.43	-1.53									
CHD7	*								-1.75						1.62	1.08		
HAUS6	*		1.05				-1.03	-1.80	-1.97						-1.10			
IRS1	*																-1.92	
ITPRIPL1	*				1.31		-1.74								-1.32			
KAT7	*							-1.58	-1.60									
LRRFIP1	*		-1.14		1.02			-1.26	-1.68	-1.09					-1.04	-1.31		
NUDCD1	***																	
PKMYT1	*							-1.15		1.20								1.02
POLR2B	*							-1.11	-1.15									
PRMT7	*						-1.07	-1.65	-1.64						-1.04			
RRM2	***		-1.07	1.02	1.78		-1.29	-1.89	-2.38				-1.13	-1.92			2.46	2.82
S100A11	***						-1.37	-2.07	-1.87				-1.36	-1.43	-1.13			
STAT5A	**													1.55	1.99			
TBCB	*		-1.06					-2.09	-2.23	-1.27			-1.45	-1.84	-1.59			
UHRF1	*	-1.68	1.01	2.23	2.28		-1.18	-1.27	-1.45							1.29	2.62	2.77
UTP20	*		1.38	1.50	1.35									1.06	1.04			
UTP3	*		1.03															

Tfh, follicular helper T cell; Th, T helper cell; Treg, regulatory T cell; TAM, tumor-associated-macrophage; NK, natural killer cell; DC, dendritic cell; None, correlation without adjustment; Purity, correlation adjusted for tumor purity; Cor, R value of Spearman’s correlation. *P < 0.01; **P < 0.001; ***P < 0.0001, ****P < 0.0001.

### Upregulated Proinflammatory Cytokines Such as TNFα, IL6, CSF1 and CSF3, Trained Immunity (TI) Marker IL-32, and TI Metabolic Enzymes, and Epigenetic Reprogramming Enzymes Indicate TI in EC Infected by MERS-CoV and Drive Cytokine Storm; and Upregulated CSF1 and CSF3 Demonstrate a Novel Function of EC in Promoting Myelopoiesis

**Figure 4:**
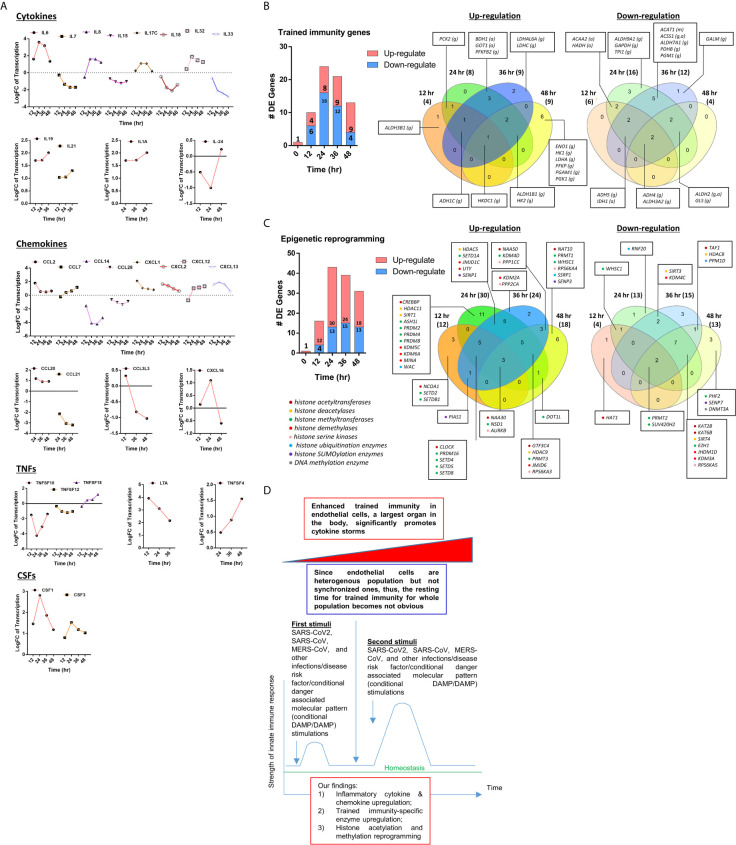
**(A)** The 31 cytokines, chemokines, tumor necrosis factor family members (TNFs), and colony stimulation factors (CSFs) are differentially expressed (DE) in human microvascular endothelial cells (HMECs) infected by MERS-CoV at different HPI (hours post infection). These secretory molecules were selected to be presented since their expressions were changed with the P value <0.05 and |logFC|>1 at more than one time point. **(B)** As coronavirus infection progress from 12 hours (h), 24h, 36h, and 48h post infection (PI), more trained immunity pathway enzymes show upregulation than the controls in time-course sensitive manner in human microvascular endothelial cells (HMECs) infected by MERS-CoV. These trained immunity pathway enzymes were selected to be presented since their expressions were changed with the P value <0.05 and |logFC|>1 (see **Supplementary Table 4**); **(C)** As coronavirus infection progress from 24h and 36h post infection (PI), more out of 168 epigenetic reprogramming enzymes are upregulated than 12h and 48h PI in human microvascular endothelial cells (HMECs) infected by MERS-CoV. These epigenetic enzymes were selected to be presented since their expressions were changed with the P value <0.05 and |logFC|>1. The color in front of each gene represents the enzyme category it belongs to (see **Supplementary Table 5**). **(D)** A new working model: Infection of vascular endothelial cells by SARS-CoV2/MERS-CoV causes endothelial cell trained immunity induces cytokine storm.

We critically analyzed EC immunity evidence and found that immunometabolism and innate immune memory (trained immunity, TI) enhance the innate immune functions of ECs (8, 44) following a priming-resting-rechallenging with CVD risk factors, PAMPs and DAMPs. A previous report showed that endothelial cells are central regulators for suppressing cytokine amplification and cytokine storm (117) during influenza virus infection (118), which was correlated with our previous report on tolerogenic function of ECs (37). On the other hand, our recent paper reported novel molecular evidence that human aortic EC have innate immune memory (also termed trained immunity, TI) function and that acetylation of histone 3 lysine 14 (H3K14) bound in the genomic regions that encode TI enzymes in oxidized low-density lipoprotein (oxLDL)-derived proatherogenic lipids LPC-activated human aortic EC are increased in comparison to that encoded EC activation genes (33). Along the same line, a recent report also showed that oxLDL-mediated EC activation represents an immunologic memory event, which triggers metabolic and epigenetic reprogramming (119). Several cytokines have been reported as the readouts of TI such as TNFα, IL-1β, IL-6 and IL-32 (44), in which IL-32 is a marker of TI (120). To search for the evidence that TI as a mechanism to enhance EC responses infected by MERS-CoV, the expression changes of cytokines and chemokines were analyzed in MERS-CoV-infected human microvascular EC (HMEC) microarray datasets with a time course of 0h, 12h, 24h, 36h and 48h PI. As shown in **Figure 4A**, the expressions of 20 cytokines and chemokines such as IL-6, IL-8, IL-17C, IL-32, IL-19, IL21, IL-1A, IL-24, TNFSF18, TNFSF4, CSF1, CSF3, CCL2, CCL7, CXCL1, CXCL2, CXCL12, CXCL13, CCL20, and CXCL16 were significantly upregulated. TNFSF4 has been reported to be upregulated in metabolic reprogramming in macrophages stimulated with immune complex (ova-IC) (121). CSF1 is upregulated in TI (122), and CSF3 secreted from EC is a major source to promote myelopoiesis during systemic inflammation (123). In addition, as shown in **Table 3**, 15 cytokine and chemokine receptors including IL3RA, IL4R, IL7R, IL13RA2, IL15RA, IL17RE, IL20RB, CCRL1, CXCR4, TNFRSF10A, TNFRSF10B, TNFRSF10C, TNFRSF10D, TNFRSF11A, IFNGR2 were upregulated in in MERS-CoV-infected HMEC. Similar to TNFSF4, TNFSF10, a ligand for TNFRSF10A, TNFRSF10B, TNFRSF10C, TNFRSF10D, has been reported to be upregulated in metabolic reprogramming in macrophages stimulated with immune complex (Ova-IC) (121) and in neutrophils stimulated by a prototypic TI stimulus BCG vaccine for EC adhesion (124). Taken together, these results have demonstrated that at least 20 cytokines and chemokines are selectively upregulated as MERS-CoV infection process progresses, suggesting the enhancement of innate immune responses as one of the key features of TI. The upregulation of TI marker cytokine IL-32 further supports this argument. High levels of expressions of cytokines such as IL6, IL19, IL21, IL1A, CSF1 and CSF3 in EC after MERS-CoV infection are 12 hours after infection, earlier than that of most cytokine and chemokine receptors at 24 hours after infection, emphasizing the forward signaling roles of ECs in stimulating migrated inflammatory cells. Meanwhile, an upregulation of the cytokine receptors TNFRSF10A, TNFRSF10B, TNFRSF10C, and TNFRSF10D comes together with a down expression level of the ligand, TNFSF10, suggesting there may be a reverse signaling delivered from other cells to further stimulate the activated ECs. These results indicate the highly productive cellular interaction between activated ECs and migrated inflammatory cells. Furthermore, upregulations of CSF1 and CSF3 in ECs during infection have demonstrated EC promotion of myelopoiesis to accelerate the maturation and roles of myeloid cells in anti-infectious diseases, which is a new innate immune function of ECs (1, 8) beyond promoting the inflammatory cell migration (63), and is a function usually carried out by hematopoietic stem cell (HSC) niche supportive cells (123). We reported that IL-17A stimulation of human aortic ECs upregulates CSF2 (granulocyte-macrophage colony stimulating factor, GM-CSF) (31), which stimulates myelopoiesis and TI in human monocytes (125).

**Table 3:** The receptors of cytokines, chemokines, TNFs, and IFNs are differentially expressed in HMECs infected by MERS-CoV at 12 hours (h), 24h, 36h, 48h post infection (PI) with ic-MERS virus.

Gene symbol	Binding Ligand	logFC			
		12(hr)	24(hr)	36(hr)	48(hr)
*Cytokine receptor*					
IL3RA	IL-3/CSF-2/IL-5	-0.26	1.01	0.25	-0.35
IL4R	IL-4/IL-13	1.03	0.94	0.73	0.64
IL7R	IL-2/IL-4/IL-9/IL-15		1.96	1.65	1.17
IL13RA2	IL-13	-0.23	1.66	0.73	0.68
IL15RA	IL-15/IL-2	2.93	2.54	-0.64	0.95
IL17RE	IL-17	0.84		1.16	
IL20RB	IL-19/IL-20/IL-24		1.62	0.54	0.43
IL1R1	IL-1A/IL-1B/IL-1RA	-0.87	-2.81	-2.93	-2.55
IL6ST	IL-6 family	-1.01	-2.17	-2.39	-1.92
IL10RB	IL-10	-0.53	-1.53	-1.36	-0.60
IL13RA1	IL-4/IL-13	-0.28	-0.51	-1.11	-1.45
IL17RA	IL-17A	-0.22	-1.08	-0.88	
*Chemokine receptor*					
CCRL1	CCL19/CCL21/CCL25			1.96	0.94
CXCR4	CXCL12	-0.17		-0.22	1.17
CCR6	CCL20			-0.95	-1.21
*TNF receptor*					
TNFRSF10A	TNFSF10		1.05	1.29	1.60
TNFRSF10B	TNFSF10	1.52	1.79	1.52	0.46
TNFRSF10C	TNFSF10	2.56	3.54	3.22	1.81
TNFRSF10D	TNFSF10		1.83	1.77	0.58
TNFRSF11A	TNFSF11	1.96	4.23	4.45	2.75
TNFRSF11B	TNFSF11	-1.00	-1.63	-1.00	
TNFRSF12A	TNFSF12	0.89		-1.02	-0.92
CD40	CD40L	-0.31	-1.80	-1.82	-1.05
TNFRSF4	OX40L	0.44	-1.18	-1.15	-1.40
TNFRSF14	TNFSF14	0.55		-0.59	-1.76
TNFRSF19	TNFSF19	0.35	-1.09	-0.71	-0.51
TNFRSF25	TNFSF25	-0.48	-1.24	-0.82	-0.25
*IFN receptor*					
IFNGR2	IFNG	0.53	1.36	1.27	0.71
IFNAR1	IFNA	-0.57	-1.12	-1.03	
IFNGR1	IFNG		-1.07	-1.40	-0.72

These findings also suggest that EC upregulation of CSFs in promoting myelopoiesis is another indicator of TI function of ECs as reported (126). Previous reports that microbial components in priming and inducing TI are limited to bacterial and fungal compounds such as lipopolysaccharide (LPS), Bacille Calmette-Guérin vaccine (BCG), β-glucan; and that viral proteins have not yet been reported (44). Our novel results have demonstrated that MERS-CoV infection of ECs is also capable of inducing TI. Previous reports also indicated that the resting time between priming stimulation and re-stimulation during the TI can range from 24 hours to 6 days *in vitro* cell culture models (127). Continuous upregulation of many proinflammatory cytokines and innate immune effectors during 48 hours PI may be driven by the TI mechanism because the following arguments: *1)* human microvascular ECs are a heterogenous population so that the cells may be different in resting time requirement; *2)* no special regulators are identified for the resting time during TI; and *3)* no essential mechanisms have been identified to correlate the length of resting time with the enhancement scale of proinflammatory response after re-stimulation (44).

To further search for the evidence of TI in HMECs infected with MERS-CoV, the expressions of 95 TI genes were examined in **Figure 4B** as we reported (33). Four glycolysis enzymes were upregulated at 12h PI, eight were upregulated at 24 and 36 hr PI, and nine glycolysis enzymes including hexokinase 1 (HK1, a rate-limiting enzyme (128) to phosphorylate glucose to produce glucose-6-phosphate, the first step in most glucose metabolism pathways) were upregulated in at 48h PI (*p* <0.05). Two acetyl-CoA generating enzymes BDH1 and GOT1 were upregulated too. With the input of TI genes with p value<0.05, the pathway analysis indicated glycolysis was inhibited at 12 and 24 hr post MERS infection, but reversed at 48 hr PI (**Supplementary Figure 1**). Among the genes, those involving in the glycoses pathway, ENO1, PGAM1 and PGK1 were greatly induced at 48hr PI, compared to the quiescent or suppressed status at earlier time points. Of note, In SARS-COV-2 infected Caco-2 cells, the induction of ENO1 was observed at 24hr PI at the translation level, as well as the protein level (129). Of note, the expressions of mevalonate biosynthesis enzymes were not modulated. On the other side, epigenetic reprogramming has been reported as the key component for TI (33, 44). We reported that more downregulation than upregulation of 164 histone modification enzymes in metabolic diseases makes a few upregulated enzymes the potential novel therapeutic targets in metabolic diseases and other inflammatory diseases (62). Since DNA methylation also participates in TI in addition to enzymes in histone acetylation and methylation (130), thus, 168 epigenetic reprogramming genes (ERGs) in eight groups including 164 histone modification enzymes such as histone acetyltransferases, histone deacetylases, histone methyltransferases, histone demethylases, histone serine kinases, histone ubiquitination enzymes, histone small ubiquitin-like modifier (SUMO)ylation enzymes plus four DNA methyltransferases were examined in **Figure 4C**. One ERGs, 12 ERGs, 30 ERGs, 24 ERGs and 18 ERGs were upregulated in 0h, 12h, 24h, 36h, and 48h PI (fold changes = | log2|>1, *p* <0.05), respectively. In the detailed results, as shown in **Table 4A**, 15 out of 31 histone acetyltransferases (48.4%) and five out of 18 histone deacetylases (27.8%) were upregulated in coronavirus infections. Among them, seven histone acetyltransferases and four histone deacetylases were increased in HMECs infected by MERS-CoV. As shown in **Table 4B**, 26 out of 55 histone methyltransferases (47.2%) were upregulated in coronavirus infections. 11 out of 24 histone demethylases (45.8%) were upregulated in coronavirus infections. Sixten histone methyltransferases and eight histone demethylases were increased in HMECs infected by MERS-CoV. Of note, future work is needed to determine specific requirements for histone methylation and acetylation in coronavirus-infected human EC as we reported for histone 3 lysine 14 acetylation in LPC-activated human aortic EC using mass spectrometer and chromatin immunoprecipitation followed by DNA-sequencing (CHIP-Seq) (34). Taken together, considering that the endothelium is a highly specialized, dynamic, disseminated organ composed of 1 to 6 × 10^13^ ECs covering a surface area of more than 1000 square meters (1, 8), our findings have provided novel transcriptomic insights on epigenetic reprogramming in human ECs infected by MERS-CoV and lung cells infected by SARS-CoV, suggesting that TI is a significant mechanism for enhancement of endothelial contributions of cytokine storms and thromboembolism to COVID-19 (SARS-CoV2-induced disease) (131) (**Figure 4D**). Our findings were well correlated with recent proposals that TI is going to be targeted for reducing susceptibility to and the severity if SARS-CoV2 infection (132).

**Table 4A:** The 31 histone acetyl transferases and 18 deacety lasesare differentially expressed in endothelial cells and other cells infected by several coronaviruses such as SARS-CoV, MERS-CoV and mutant coronaviruses.

Virus	MERS-Cov				SARS-Cov					SARS-Cov (ORF6 mutant)					MA-15 (SARS-CoV)		
Origin	HMECs				Calu-3 cells										Mouse Lung tissues (WT)		
Time	12(hr)	24(hr)	36(hr)	48(hr)	12(hr)	24(hr)	36(hr)	48(hr)	72(hr)	12(hr)	24(hr)	36(hr)	48(hr)	72(hr)	2(d)	4(d)	7(d)
*Histone acetylation*								-1.08							1.04		
HAT1	-1.27																
KAT2A							-1.11										
KAT2B		-1.66	-2.30	-2.19				-1.46	-1.05			-1.05					
KAT6A													1.59	1.92			
KAT6B		-1.04	-1.34	-1.07													
KAT7							-1.58	-1.60									
KAT8							-1.53	-1.48									
ELP3							-1.09	-1.18									
NCOA1	1.06						-1.02										
NCOA3							-1.03	-1.05					1.04				
CLOCK	1.21	2.06	1.24				-1.40	-1.61									
CREBBP		1.40						1.30					1.86	1.92			
EP300													1.63	1.59			
ATAT1							-1.22	-1.52	-1.34			-1.34	-1.02	-1.58			
TAF1			-1.36														
GTF3C4		1.31	1.38	1.22			-1.35	-1.66	-1.17			-1.17	1.31	1.52			
NAT10				1.18													
NAT9							-1.58	-1.39									
NAT8									-1.31			-1.31		-1.18	-1.18		
NAT14							-1.69	-1.73									
BPTF								-1.24					1.20				
NAA10							-1.67	-1.65					-1.14				
NAA20						-1.12	-1.66	-1.81					-1.04		1.07		
NAA30	1.01	1.40	1.05	1.64			-1.01	-1.46									
NAA60																	
NAA40							-1.14	-1.79									
NAA50			1.31	1.13													
TAF5L							-1.23	-1.11					1.11	1.10			
TAF6L							-1.34	-1.31									
BTAF1													2.03	1.79			
*Histone deacetylation*																	
HDAC4								-1.48									
HDAC5		1.09	1.16					-1.16	-1.05			-1.05					
HDAC7							-1.02										
HDAC9		3.24	5.01	2.09			1.79	3.12	2.99			2.99	3.19	2.85			
HDAC6							-1.75	-1.68	-1.44			-1.44		-1.07			
HDAC10							-1.30										
HDAC1						-1.09	-2.00	-2.17	-1.35			-1.35	-1.28	-1.28			
HDAC2							-1.19										
HDAC3							-1.11										
HDAC8			-1.00				-1.77	-1.34							-1.17		
SIRT1		1.10											2.08	2.30			
SIRT2							-2.02	-2.18	-1.28			-1.28					
SIRT3		-1.06	-1.17			-1.44	-1.82										
SIRT4		-1.21	-1.66	-1.27			-1.06							1.68			
SIRT5							-1.54	-2.09					-1.30	-1.04			
SIRT6							-1.54	-1.57									
SIRT7							-1.06										
HDAC11		2.36					-1.61	-2.00	-1.01			-1.01	-1.45		-1.71		

The Log FC value of genes with P value < 0.05 and |logFC|>1 are shown.

**Table 4B:** The 55 histone methyltransferases and 24 demethylases are differentially expressed in endothelial cells and other cells infected by several coronaviruses such as SARS-CoV, MERS-CoV and mutant coronaviruses.

Virus	MERS-Cov				SARS-Cov					SARS-Cov (ORF6 mutant)					MA-15 (SARS-CoV)		
Origin	HMECs				Calu-3 cells										Mouse Lung tissues		
Time	12(hr)	24(hr)	36(hr)	48(hr)	12(hr)	24(hr)	36(hr)	48(hr)	72(hr)	12(hr)	24(hr)	36(hr)	48(hr)	72(hr)	2(d)	4(d)	7(d)
*Histone methyltransferase*																	
KMT2A												1.12	1.67	1.98			
KMT2D						-1.10		-1.52									
KMT2C								1.05				1.58	1.45	2.11			
KMT2B													1.77	1.40			
KMT2E												1.25	1.09	1.91			
SETD1A		1.21	1.06										1.20				
SETD1B													1.10				
ASH1L		1.13										1.30	1.59	1.80			
EHMT2							-1.95	-1.68				-1.01	-1.10				
SUV39H1							-1.41	-1.17									
SUV39H2							-1.03	-1.45	1.15								
SETDB1	1.28												1.25				
EHMT1							-1.17	-1.03									
PRDM2		1.40	-1.36	-1.15			-1.18	-1.24	-1.53			1.07	1.95	1.61			
SETD2	1.19											1.03	1.59	1.46			
NSD1	1.46	1.43	1.66	1.17				-2.53	-1.73								
SYMD2																	
DOT1L		1.16		1.20		-1.04		-1.30				1.15	1.45	1.62			
SETD7						-1.07	-1.57	-1.97									
SETD8	1.40	1.10	1.03														
SUV420H1																	
SUV420H2	-1.44	-2.24	-1.65	-1.20												-3.08	-2.21
EZH2																1.04	1.50
EZH1		-1.61	-1.61	-1.03			-1.85	-1.52									
SETD3							-1.10	-1.32									
SETD4	1.61	2.48	2.07				-1.05	-1.01									
SETD5	1.18	1.16	1.09									1.02	1.51	1.11			
SETD6-1																	
WHSC1	-1.13	-1.65		1.20			-1.60	-1.79					-2.15				1.23
SETMAR								-1.49									
SETDB2							-1.09	-1.67	1.24								
PRDM16	1.30	1.66	1.56														
PRDM5							-1.03										
PRDM1							1.33	2.96	2.80			1.58	3.51	4.06			
PRDM11												1.17	1.89	1.42			
PRDM6								-1.26							-1.06		
PRDM12						-2.03	-2.44	-2.63	-2.28			-1.49	-1.05				
PRDM14																	
PRDM7																	
PRDM9																	
PRDM8		2.00						-1.71							-1.70		
PRDM4		1.02										1.47	1.92	1.44			
PRDM10																	
PRDM15																	
PRMT2	-1.29	-1.59	-1.40	-1.03			-2.04	-2.05	-1.71			-1.38	-1.35	-1.71			
PRMT6							-1.60	-1.48									
PRMT3		1.38	1.35	1.25													
PRMT1				1.41													
PRMT8																	
CARM1																	
PRMT7						-1.07	-1.65	-1.64					-1.04				
SMYD1																	
SMYD3							-1.66	-1.90	-1.27					-1.10	-1.34		
SMYD4							-1.19	-1.41					-1.65				
SMYD5							-1.41	-1.08									
*Histone demethylase*																	
KDM1A						-1.04	-1.44	-1.49									
KDM1B							-1.07	-1.95									
KDM2A			1.13	1.71										1.93			
KDM2B																	
KDM3A		-1.67	-1.63	-1.40			-1.17	-1.06					1.02				
KDM3B						-1.05	-1.50	-1.80									
KDM5A																	
KDM5B							-1.06	-1.05					1.50	1.42			
KDM5C		1.05					-1.54	-1.14									
KDM5D						-1.09		-1.63									
KDM4A							-1.11										
KDM4B																	
KDM4C		-1.33	-1.58					-1.25	-1.07			1.77	1.03				
KDM4D			1.89	1.13			-1.34	-1.12									
KDM6A		1.32										1.43	1.97	1.99			
JHDM1D		-1.30	-1.29	-1.17													
UTY		2.48	1.82				2.95					2.49					
JMJD1C	1.88	1.75	1.08	1.03	1.10	1.68	3.01										
KDM8							-1.47	-1.77	-1.32								
JMJD6		2.38	2.15	1.50			-1.03						2.09	1.65			
MINA		1.24															
C14ORF169								-1.02									
PHF8												1.39	2.29	2.46			
PHF2								-1.11									

The LogFC value of genes with P value <0.05 and |logFC| >1 are shown.

### Increased Unfolded Protein Response and ER Stress, Downregulated Mitochondrial Oxidative Phosphorylation Complexes and Increased Reactive Oxygen Species as the Signal Mechanisms Facilitate Proinflammatory Response and TI

**Figure 5:**
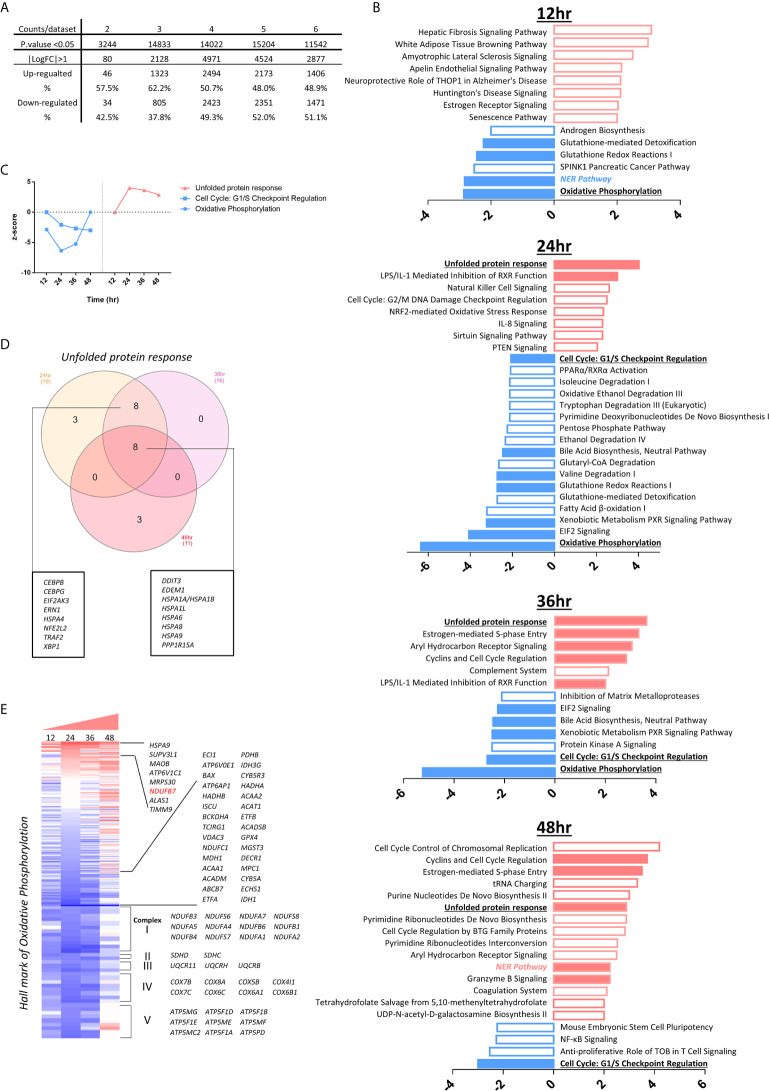
The modulations of innate immune regulatomic genes in HMECs infected with MERS-CoV indicate significant signal pathway changes including increased unfolded protein response, promoted endoplasmic reticulum (ER) stress, enhanced reactive oxygen species (ROS), and downregulated mitochondrial oxidative phosphorylation complexes, which may facilitate proinflammatory response and trained immunity (TI). **(A)** The total counts of genes those are differential expressed in dataset 2-6 with *P* value<0.05 and with |logFC|>1. **(B)** Ingenuity Pathway Analysis identified the pathways with |*z* score|≥2 in each time point. Bars in red represent activation of the pathway and in blue represent inhibition. Empty bars are pathways only identified in each time point, and filled ones are pathways shared by at least two time points. Specifically, three pathways underlined are shared by three time points. **(C)** The dot plot described the trend of the *z* scores of these pathways (*z*-score=0 means no significance). **(D)** Vein diagram shows the overlap of the genes in three datasets (24h, 36h and 48h PI) those are enrolling in the activation of unfolded protein response. **(E)** Oxidative phosphorylation are inhibited upon icMERS infection at 12 hours (h), 24h, and 36h post infection (PI). To further understand the differential expression genes related to OXPHOS, we generate the heatmap for 200 hall marks of OXPHOS from GSEA hall mark datasets, including genes from mitochondrial complex I-IV and others. Genes with |logFC|≥1 are listed.

SARS-CoV2 virus, the etiological agent of coronavirus disease 2019 (COVID-19), can lead to COVID-associated coagulopathy (CAC) based on evidence of microthrombi and macrothrombi, both in venous and arterial systems (57, 133), increased levels of inflammatory mediators, EC dysfunction, and infiltration of inflammatory cells into the organs (134). To improve our understanding on SARS-CoV2, we chose MERS-CoV-infected ECs as a model system since the genome of MERS-CoV has as high as 50% homology to that of SARS-CoV2 (135). This time we included all differential expressed genes at 0h, 12h, 24, 36, 48h post-infection (PI) (in the datasets 2-6 with *p* <0.05), respectively (**Figure 5A**). We then performed the IPA on the differential expressed genes and identified the pathways with |***z*** score|≥2 in each time point. Bars in red represent activation of the pathway and in blue represent inhibition (**Figure 5B**). Empty bars were pathways only identified in each time point, and filled ones were pathways shared by at least two time points. Specifically, three pathways underlined, unfolded protein response (UPR), cell cycle: G1/S checkpoint regulation, oxidative phosphorylation were shared by three time points. In **Figure 5C**, the dot plot described the trend of the ***z*** scores of these pathways, of which the pathway of URP was activated at 24h, 36h and 48h PI. It has been reported that UPR and ER stress have crucial functions in immunity, inflammation (136) and provoke many diseases, including autoimmunity (137). In **Figure 5D**, the Vein diagram showed the overlap of the genes in three datasets, which are involved in the activation of UPR (138). Virus infection represents an arm race between virus and the host. On the one hand, the host mobilizes the UPR in an attempt to restrict virus infection since UPR as a consequence of ER stress results in activation of protein kinases IKKβ and JNK, impacting TLR signaling and immune training (139). On the other hand, the virus subverts or even manipulates the UPR to assist in its own infection. The consequence of this is that the UPR is often skewed during virus infections to either favor virus elimination or virus invasion. ER stress has been found to affect NLRP3 inflammasome activation (5) via UPR, calcium or lipid metabolism and reactive oxygen species (140). UPR and ER stress are mediated in part by the assembly of multi-protein complex, named the UPRosome, which regulates the crosstalk with other pathways and triggers adaptive programs or apoptosis of terminally damaged cells (141).

To understand the differential expression genes related to mitochondrial oxidative phosphorylation (OXPHOS), we generated the heatmap for 200 hallmark genes of OXPHOS from the gene set enrichment analysis (GSEA) database (https://www.gsea-msigdb.org/gsea/index.jsp) hallmark datasets. Besides the mitochondrial complex genes, the rest of the hallmark genes were ordered according to the value of logFC in 24 hr, which has the absolute highest Z score of OXPHOS pathway. Genes with |logFC|≥1 were listed. As shown in **Figure 5E**, MERS-CoV infection downregulated the OXPHOS-related long list of mitochondrial complexes (I, II, III, IV and V) genes, especially at 12h, 24h, and 36h PI, respectively. Of note, we matched the genes to the IPA analysis database, found that downregulated genes encoding mitochondrial complex I-V were the major genes, deciding the inhibition of OXPHOS. At 48h PI, the inhibition of OXPHOS was not obvious. Our findings were well correlated with the reports that a broken Krebs cycle leads to the accumulation of two metabolites, citrate and succinate, both of which triggers proinflammatory response (142) and trained immunity (143).

### Increase of the Cell Death Regulators Such as Mitotic Catastrophe-Regulated Cell Death, Apoptosis, Ferroptosis and Inflammasomes-Driven Pyroptosis in Endothelial Cells Infected With MERS-CoV May Trigger Thrombosis, Which Is Partially Suppressed by BRD4 Inhibitor JQ1; and Upregulated Coagulation Factors and Protease-Activated Receptors (PARs) and Downregulated Anticoagulants Also Promote Thrombosis Potential

**Figure 6:**
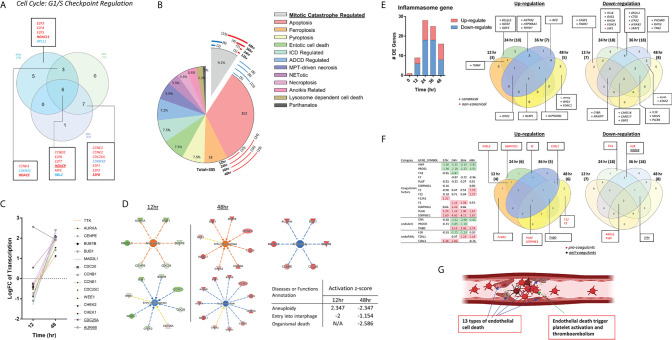
Sustained inhibition of cell cycle: G1/S checkpoint regulation pathway and a cell pathway switch along time course indicates an abnormal EC replication upon MERS-CoV infection. **(A)** Vein diagram shows the overlap of the genes in three datasets (24hr, 36hr and 48hr) those are enrolling in the activation of Cell Cycle: G1/S Checkpoint Regulation. (Genes underlined in 6A are overlapped with genes those significantly reversed by JQ1 (see data analysis from GSE53999 in **Supplementary Table 6** and **Supplementary Figure 2**). **(B)** Pie chart show the annotation of cell death related gene from 13 categories. Numbers in each pie are the percentage of genes belong to this category. Of note, the response of genes from Mitotic Catastrophe Regulated cell death show remarkable time course-sensitivity. The length of curve represent the percentage of genes with |logFC|>1 in the category, Red, means upregulated and Blue, means downregulated (see **Supplementary Table 7**). The change of genes enrolled in apoptosis reached the highest in 24h PI (**Supplementary Figure 3**). **(C)** Transcription change of genes from category of Mitotic Catastrophe Regulated at 12 and 48 HPI. **(D)** Disease and Function prediction generated from IPA based on the gene change of Mitotic Catastrophe Regulated. Z- scores are included in the table. No significance were found in 24 and 36 HPI. **(E)** The expressions of inflammasome pathway regulators were modulated in HMEC infected by MERS-CoV, suggesting that inflammatory cell death (pyroptosis) contribute to endothelial cells infected by MERS-CoV (see **Supplementary Table 8**). **(F)** The expressions of 12 pro-coagulation factors were upregulated and the expressions of two anti-coagulation factors were downregulated in HMECs infected by MERS-CoV. **(G)** Endothelial cell death contributed by 13 cell death pathways may trigger thromboembolism. The part of figure was created with BioRender.com.

A new Nature Review suggests that the prevalence of cardiovascular comorbidities in patients with COVID-19 in the world reaches as high as 19.2-25% (144). It brought us to the attention of the complications associated with unhealthy cell cycle and cell death of vascular ECs during virus infection. In **Figure 6A**, cell-cycle: G1/S checkpoint regulation was inhibited in HMECs at 24h, 36h, 48h PI by MERS-CoV, indicating a failure of checking and repairing DNA damage before replication (145, 146), in which the induction of E2F transcription family and cyclin family plays a major role. Moreover, to further examine EC death caused by coronavirus infection in details, comprehensive cell death pathways were examined (60, 147, 148). We hypothesized that cell death regulatomic genes are modulated in HMECs infected with MERS-CoV. To test this hypothesis, we examined the expression changes of 305 cell death regulatomic genes in the 13 types of cell death pathways in a new international nomenclature (149) as we reported previously (150). In **Figure 6B** and **Table 5**, we listed the differential expressed cell death regulators in 13 categories (151), among which enrolled in apoptosis reached the highest in 24h PI. Of note, we observed that genes in mitotic catastrophe regulated pathway were reduced at the early time and were remarkably induced with the progress of infection (**Figures 6B–D**), projecting a significant aneuploidy in ECs at 12h PI (z-score>2) and inhibition of aneuploidy and organismal death at 48h PI (z-score<-2). Moreover, the regulators for eight types of cell death such as apoptosis (**Supplementary Figure 2**), MPT-driven necrosis, ferroptosis, pyroptosis, entotic cell death, ADCD regulated, mitotic catastrophe regulated, and anoikis regulated showed more modulations (modulations >3 genes) than others. Since many types of cell death are newly characterized, the potential causative effects of certain new types of EC death in promoting thrombosis will be determined in the future.

**Table 5:** The expressions of the regulators of all 13 cell death Cell types were modulated in human microvascular endothelial cells infected by MERS-CoV.

Categories	12hr		24hr		36hr		48hr	
	↑	↓	↑	↓	↑	↓	↑	↓
Mitotic Catastrophe Regulated (28)	1	2	1	6	4		15	
Apoptosis (102)	10	5	24	15	16	8	19	8
Ferroptosis (24)		2	5	5	3	5	4	4
Pyroptosis (23)	1	2	1	2	1	3	1	1
Entotic cell death (23)		1	2	4	1	3	3	3
ICD Regulated (23)		1					1	
ADCD Regulated (22)	1	2	1	6	1	6	1	4
MPT-driven necrosis (18)	1		3	3		3		3
NETotic (12)		1	2	1	1	1		
Necroptosis (10)	1		2	1	1	2		1
Anoikis Related (10)		1		3	1	2	1	2
Lysosome dependent cell death (7)	1		1	2	1	1	1	1
Parthanatos (3)				1				

Inflammasomes serve as the sensors for PAMPs and DAMPs (9) and bridge infections and metabolism-derived DAMPs to inflammation as our new model proposed (5, 140) and reported (13, 18, 20–22, 25, 152, 153). To detail the expression changes of pyroptosis (inflammatory cell death) pathways in EC, we examined the expression of 94 inflammasome regulators in HMEC infected by MERS-CoV, which were collected from the KEGG database inflammasome pathway (KEGG Pathways NOD-Like Signaling Pathways.). The KEGG inflammasome pathway gene list contains both canonical and non-canonical inflammasome/gasdermin D pathway regulators (154). As shown in **Figure 6E**, three, 10, seven and five inflammasome regulators were upregulated in 12h, 24h, 36h and 48h PI in HMEC infected by MERS-CoV, respectively. In addition, seven, 18, 18, and eight inflammasome regulators were downregulated in 12h, 24h, 36h and 48h PI in HMEC infected by MERS-CoV, respectively. Moreover, in addition to modulation of canonical inflammasome pathway regulators, one non-canonical inflammasome pathway regulator GBP3 was also upregulated at 24 h PI; and two, two and one non-canonical inflammasome regulators were downregulated at 24h, 36h, and 48h PI, respectively. These results suggest that inflammasome regulators are significantly modulated in EC infected by MERS-CoV to mediate EC pyroptosis.

We then examined a hypothesis that the upregulation of cell death regulatomic genes are associated with modulation of prothrombotic regulators in EC. Nine coagulation factors, one anticoagulants, and three protease-activated receptors (PARs) out of 35 coagulation regulators (155) were upregulated, and two anticoagulants were downregulated (**Figure 6F**). These results have demonstrated that coronavirus infection of ECs not only increases cell death of EC but also upregulates thrombosis potential (155, 156), which are correlated well with that reported (157). Our results have demonstrated that EC death contributed by 13 cell death pathways together with increased thrombogenic regulators may trigger thromboembolism, which may be an important mechanism underlying increased cardiovascular complications of COVID-19 (**Figure 6G**).

The bromo- and extra-terminal domain (BET) signaling pathway plays an important role in cell proliferation, cancers, immune responses, and pro-inflammatory responses (158). BRD4, a member of BET protein family, has been reported in cancer for its role in super-enhancers (SEs) organization and oncogenes expression regulation (159). We hypothesized that BRD4 plays a significant role in mediating various cell death pathways related to inflammation in HMEC infected by MERS-CoV. In a microarray datasets in the NIH-NCBI-GeoDatasets database, #GSE53999, the pathway analysis indicated that pathway of Cell Cycle: G1/S Checkpoint Regulation was significantly activated upon JQ1 (BRD4 inhibitor) administration in the conditions of TNF-alpha exposure of EC (160). In **Figures 5B** and **6A**, our results indicated an sustained inhibition of cell-cycle:G1/S checkpoint regulation in HMECs at 24h, 36h, 48h PI by MERS-CoV, suggesting BRD4 inhibitor may provide therapeutic strategies for coronavirus infection through slowing the rate of G1/S phase progression in the presence of DNA damage and improving ECs repair before replication (161). Of note, by looking into the associated genes in this pathway, we found HDAC9 (62), which exhibited sustainable upregulation, could be inhibited by JQ1 (see the data analysis from GSE53999 in **Supplementary Table 6** and **Supplementary Figure 3**), highlighting a potential therapeutic target through epigenetic reprogramming.

### NRF2-Mediated ROS Regulate Innate Immune Responses, Trained Immunity, Thromboembolism, EC Activation and Death

**Figure 7:**
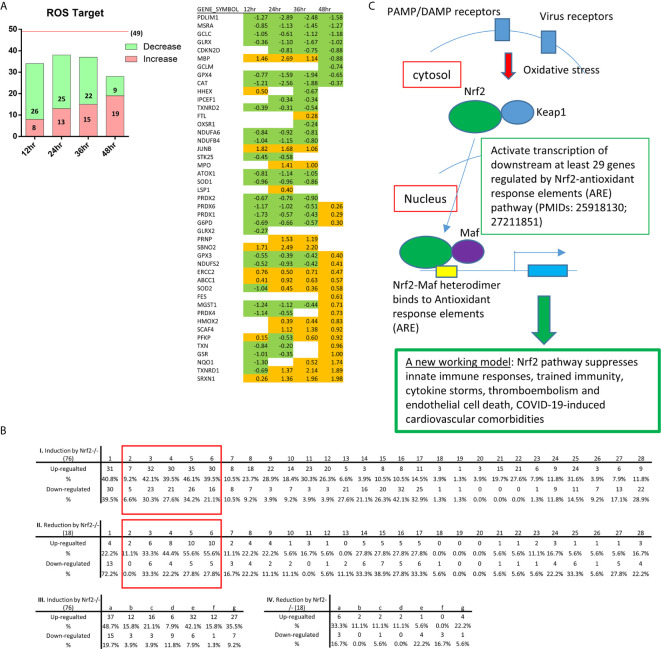
As coronavirus infection progresses in endothelial cells, the expressions of reactive oxygen species regulatomic genes are upregulated; and NRF2-suppressed reactive oxygen species (ROS) promote innate immune responses, trained immunity, thromboembolism, EC activation and death. **(A)** Bar chart show counts of ROS target genes in HMECs at 12, 24, 36, 48 hours post ic-MERS virus infection (HPI). In GSEA hall mark gene sets (HALLMARK_REACTIVE_OXYGEN_SPECIES_PATHWAY), we got 49 genes modulated by ROS. After matching to the four datasets, we selected genes with p value <0.05 and counted by Log FC, in respectively; **(B)** The Tables show the counts of NRF2 regulated genes changed in 28 endothelial datasets (I-II) and seven immune cell control datasets (III-IV). Anti-oxidative transcription factor Nrf2-regulated genes obtained for the dataset GSE7810 resulted from our analysis of gene expression in the Nrf2 deficiency (GSE7810) in comparison to that in wild-type controls. The 76 induced and 18 reduced innate immune regulatomic genes (with p value <0.05 and |Log FC|>1) were selected to match to the 28 endothelial datasets and seven immune cell datasets (also see **Table 1**). The matched genes in datasets with significant changes (with p value <0.05 and |Log FC|>1) were counted as Up and down-regulated gene numbers and the percentages were calculated, accordingly (see **Supplementary Table 9**). The red rectangle highlight the results of HMEC infected by MERS-CoV at 0, 12, 24, 36, 48 hr PI; **(C)** A new working model: Nrf2 pathway suppresses innate immune responses, trained immunity, cytokine storms, thromboembolism and endothelial cell death, COVID-19-induced cardiovascular comorbidities.

Recently we reported that mitochondrial ROS (mtROS) in human aortic EC activated by lysophosphatidylcholine (LPC) can be increased via ATP synthesis coupled and proton leak-accelerated process which differentiate physiological activation of ECs from pathological activation of ECs (27–29, 34, 162, 163). As shown in **Figure 5B**, nuclear factor-erythroid 2-related factor-2 (NRF2)-suppressed oxidative stress response (164) was found to be increased in HMECs infected by MERS-CoV, 24h PI. Among 13 cell death types listed in **Figure 6B**, ROS contribute to multiple cell death processes such as MPT-driven necrosis, ferroptosis, pyroptosis, and parthanatos (165). In addition, mitochondrial OXPHOS is connected to ROS generation as we and others reported (26, 34) and reviewed (163, 166); and ROS also mediate various signaling pathways in EC as we reviewed (140, 167). Then, we examined whether the expressions of 164 ROS regulators from the ROS hall marker genes GSEA database are modulated in HMECs infected with MERS-CoV. As shown in **Figure 7A**, the expressions of 49 ROS regulators were modulated with increased expressions of seven, 13, 15 and 19 ROS regulators in 12h, 24h, 36h, and 48h PI, respectively, suggesting that ROS play significant roles in the pathophysiological processes in HMECs infected with MERS-CoV. We then hypothesized that ROS regulates IGs expressions in various ECs. To test this hypothesis, we determined whether the expressions of IGs, modulated by various PAMPs and DAMPs in all the 28 ECs datasets and seven monocyte datasets in **Table 1**, are changed in anti-oxidative transcription factor NRF2 deficient microarray datasets NIH-NCBI-GeoDatasets database ID# GSE7810 with the method we reported (63). We argued that if the upregulation of IGs are promoted by the NRF2-mediated ROS pathway, then these genes should be induced when NRF2 is deficient (knock-out, KO) (168). As shown in **Figure 7B**, the roles of NRF2-mediated ROS in 28 ECs datasets were varied. As high as 46.1% of innate immune regulators upregulated by virus infection (datasets #2-7, **Figure 7B** boxed) were mediated by NRF2 KO. As high as 31.6% of IGs induced by DAPMs (datasets #8-28, **Figure 7B**, non-boxed) were upregulated by NRF2 KO. These results have demonstrated that NRF2-suppressed ROS pathways play significant roles in mediating innate immune responses, TI enzymes, cytokine storms (169), thromboembolism, EC activation and death in ECs stimulated by virus infections, PAMPs and DAMPs, which are well correlated with the recent clinical trials with NRF2 activators such as sulforaphane and bardoxolone methyl as potential anti-inflammatory strategy for treating COVID-19 (170–172) (**Figure 7C**).

## Discussion

The endothelium is critically important for the delivery of oxygen, nutrients, metabolites, growth factors, cytokines, hormones and other blood components throughout the body under physiological conditions (173). In 2013, we proposed a new working model that EC are innate immune cells based on our detailed analyses of EC’s capacity in carrying out 13 innate immune functions that are played by macrophages, the prototypic innate immune cell (1, 8). In our recent ATVB review, we summarized significant progress in the field (8, 174). However, several important questions remain to be addressed: *first*, how innate immune transcriptomes change in EC infected by coronaviruses; *second*, whether there is an innate immune response in endothelial cells infected by viruses and stimulated by PAMPs and DAMPs; *third*, whether upregulation of cell death regulators are associated with increased expressions of thrombogenic genes; *fourth*, whether transcriptomic analyses can show that EC have innate immune memory (trained immunity) capacity; and *finally*, whether ROS pathways play any roles in regulating innate immune responses in EC infected by viruses and stimulated by PAMPs and DAMPs. To answer these questions, we performed extensive transcriptomic analyses of a comprehensive list of 1311 IGs in more than 30 microarray datasets deposited in NIH/NCBI GeoDatasets database and the EMBL-EBI-ArrayExpress repository. We made the following findings: The majority of modulated IGs are upregulated in first 12 hours (hr) post-infection (PI), and maintained until 48 hr PI in human microvascular EC(HMECs) infected by MERS-CoV (an EC model for COVID-19). The expressions of IGs are significantly modulated in 21 human EC transcriptomic datasets by various PAMPs/DAMPs including LPS, IFNs, Notch 1 siRNAs, oxPAPC, LPC, shear stress, hyperlipidemia and oxLDL. Selective 8.1% IGs in 11 functional groups are upregulated in ECs; and upregulation of many IGs such as nucleic acid sensors are shared in EC infected by viruses and stimulated by various PAMPs and DAMPs. To address a significant issue that the prevalence of cardiovascular comorbidities in patients with COVID-19, we examined whether human EC can be infected with coronaviruses and whether coronavirus replication/transcription complexes are upregulated during coronavirus infection. Human heart EC and mouse aortic EC express all four types of coronavirus receptors such as ANPEP, CEACAM1, ACE2, DPP4 and virus entry facilitator TMPRSS2 (human heart); most of coronavirus replication-transcription protein complexes are expressed in human microvascular EC, which contribute to viremia, thromboembolism, and CVDs. We and others reported that ECs have novel innate immune memory function also termed trained immunity (TI), in which subsequent inflammation is significantly enhanced after non-specific priming. Upregulated proinflammatory cytokines such as TNFα, IL6, CSF1 and CSF3, TI marker IL-32, and TI metabolic enzymes, and epigenetic reprogramming enzymes indicate TI functional in ECs infected by MERS-CoV, which may drive cytokine storm; and upregulated CSF1 and CSF3 demonstrate a novel function of ECs in promoting myelopoiesis. Increased UPR and ER stress, downregulated mitochondrial oxidative phosphorylation complexes and increased ROS as the signal mechanisms facilitate proinflammatory response and TI. Increase of the regulators of mitotic catastrophe-regulated cell death, apoptosis, ferroptosis and inflammasomes-driven pyroptosis in ECs infected with MERS-CoV may trigger thrombosis; and upregulated coagulation factors and protease-activated receptors (PARs) and downregulated anticoagulants also promote thrombosis potential. Finally, NRF2-mediated ROS regulate innate immune responses, TI, thromboembolism, EC activation and death, which are well correlated with the recent clinical trials with NRF2 activators such as sulforaphane and bardoxolone methyl as a potential anti-inflammatory strategy for treating COVID-19 (170).

The original microarray experiments used different cells, which prevented us from comparing the effects of proinflammatory regulators in regulating the expressions of IGs in the same cell types. For example, due to the limit datasets available, we did not compare the response patterns from heterogeneous EC populations to the same stimuli. In addition, since the transcriptomic experiments were performed in many different laboratories, variations between the results cannot be ruled out due to different experimental conditions. Of note, a similar method was used in **Figure 4B** of the Nature Immunology paper from Drs. Mathis and Benoist teams (175). Although our database mining approach was not ideal, however, as the first step to fill in the important knowledge gap this approach was justified (**Table 6**). Actually, this was a common practice that we (63) and others (176) often used in studying gene expression in non-ideal. For example, transcriptomic analyses have been often performed with heterogenous peripheral blood mononuclear cell populations (PBMCs) (63) in pathophysiological conditions versus healthy conditions, which are actually composed of many cell types, such as B cells (~15 %), T cells (~70 %), monocytes (~5 %), and natural killer (NK) cells (~10 %) among others (177). Another limitation of the current study is that due to the low throughput nature of current verification techniques in the laboratories, we could not verify every result we identified with the analyses of high throughput data. We acknowledge that carefully designed *in vitro* and *in vivo* experimental models will be needed to verify the PCs and regulators deficiencies-upregulated innate IGs further and underlying mechanisms we report here.

**Table 6:** A novel research publication type with big-omics experimental database mining analyses (pioneered in our lab in 2004, PMID: 15577853) leads to original new findings and generate new hypotheses.

Category	Big-omics Database mining	Literature review	Original experimental research
Analysis of high-throughput expremental Data (Microarray from NIH-GEO-datasets.)	Yes	No	Yes (generated in the lab and not the main approach)
***New publication types after –omics and high throughput experimental data generation***	Yes	No	No
Identification of potential Targets	Yes	No	Yes
***Identification of potential Targets those were not fall into the focus of the original research***	Yes	No	No
Original new findings	Yes	No	Yes
Summary of previous reports	Yes	Yes	Yes
***More dimensions data exploring by combining multiple high-throughput datasets***	Yes	No	No
Generating new information from the public domain (NIH-GEO, Expression Atlas, GSEA and others)	Yes	No	Yes
Low throughput experimental verification	No	No	Yes
Example (IL-35 related publications from our lab)	PMID: 22438968	PMID:25387998	PMIDs: 26085094, 29371247

A few aspects of comparisons were made within this study using big-omics experimental database mining approaches, the traditional literature reviews and the original experimental-analysis.

**Figure 8:**
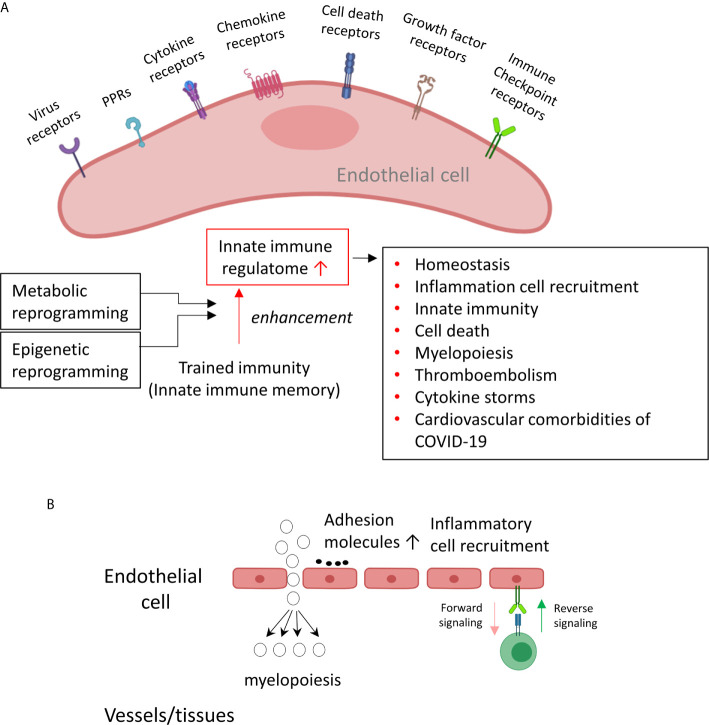
A new working model: Endothelial cells are innate immune cells, which express innate immune regulatomic genes and regulators for trained immunity-related metabolic and epigenetic reprogramming, and play significant roles in vascular homeostasis, inflammatory cell recruitment, myelopoiesis, innate immunity, cell death, thromboembolism, cytokine storms and cardiovascular comorbidities of COVID-19 and other viral infections.

To summarize our findings here, we propose a new working model (**Figure 8**). Endothelial cells are innate immune cells, which express innate immune regulatomic genes such as receptors for virus infections, DAMPs/PAMPs (PPRs), cytokines (15), chemokines (31), cell death (13), growth factors (31), immune checkpoints (37), and regulators for trained immunity (44)-related metabolic and epigenetic reprogramming (178), and play significant roles in vascular homeostasis, inflammatory cell recruitment, myelopoiesis, innate immunity, cell death, thromboembolism, cytokine storms and cardiovascular comorbidities of COVID-19 and other viral infections. We acknowledge that extensive future work is needed to verify the high-throughput results reported here with relatively low throughput methods currently in most laboratories. Nevertheless, our findings provide novel insights on the roles of upregulated innate IGs in the pathogenesis of inflammatory diseases and cancers, novel pathways underlying the multi-pathway convergent point suppression therapeutics models as well as new targets for the future therapeutic interventions for inflammations, CVDs, and cancers.

## Data Availability Statement

The datasets presented in this study can be found in online repositories. The names of the repository/repositories and accession number(s) can be found in the article/**Supplementary Material**.

## Author Contributions

YSh carried out data collections, data analyses and drafted the manuscript. JS, KX, YSu, FS, CDIV, YL, JJL, JLP, ETC, XJ, and HW provided material input. XY supervised experimental design and data analyses. XY edited the manuscript.

## Funding

Our research activities are supported by grants from the National Institutes of Health (NIH)/National Heart, Lung, and Blood Institute (HL131460, HL132399, HL138749, HL147565, HL130233, DK104116, and DK113775). 

## Disclaimer

The content in this article is solely the responsibility of the authors and does not necessarily represent the official views of the NIH.

## Conflict of Interest

The authors declare that the research was conducted in the absence of any commercial or financial relationships that could be construed as a potential conflict of interest.
