# Supplementary material for: Endothelial Immunity Trained by Coronavirus Infections, DAMP Stimulations and Regulated by Anti-Oxidant NRF2 May Contribute to Inflammations, Myelopoiesis, COVID-19 Cytokine Storms and Thromboembolism
Source: Front Immunol. 2021 Jun 25;12:653110. doi: 10.3389/fimmu.2021.653110 (PMC8269631; doi:10.3389/fimmu.2021.653110)
Supplement: Supplementary file 1 [file Image_1.pdf]

Supplementary Figure. 1

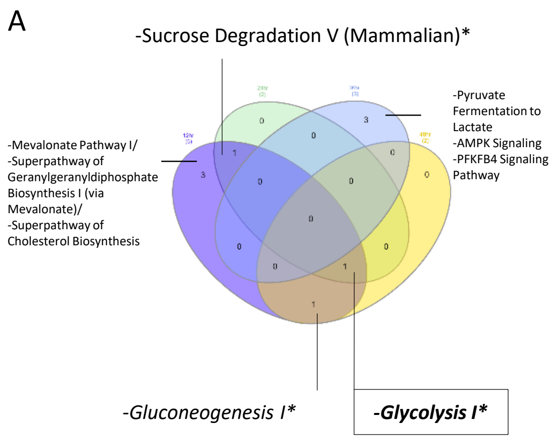

Gene Symbol	12hr	24hr	36hr	48hr	Expected
ALDOA		-0.78		0.74	↑up
ALDOB	-0.24		-0.41		↑up
ALDOC	-0.18	-0.44	-0.48		↑up
BPGM				0.56	↑up
**ENO1**			**0.85**	**1.63**	**↑up**
ENO2	0.10			0.80	↑up
ENO3		0.52	0.49		↑up
FBP1	-0.22		-0.27		↑up
GAPDH		-1.07		0.99	↑up
GPI	-0.45	-0.98	-0.62		↑up
PFKL	-0.25	-0.46	-0.57	-0.48	↑up
PFKM	-0.40		0.16	0.48	↑up
PFKP	0.15	-0.53	0.60	1.07	↑up
**PGAM1**	-0.77	-0.35	0.19	1.03	↑up
PGAM2	-0.23	-0.26		0.22	↑up
PGAM4	-0.50	0.13	0.33		↑up
**PGK1**	-0.55		0.26	1.01	↑up
PKLR	0.23		0.36		↑up
TPI1	-0.19	-1.02		0.94	↑up

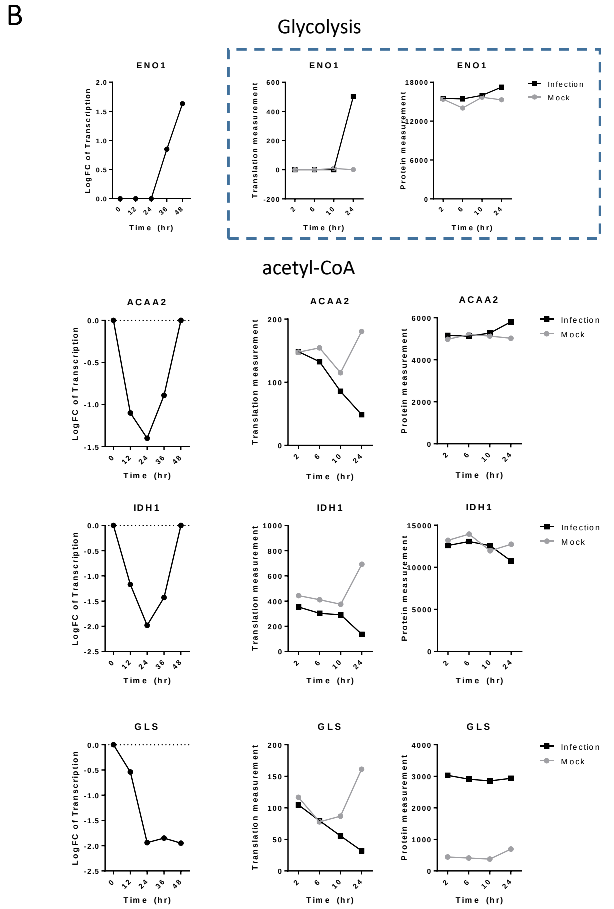

Supplementary Figure. 1

A. IPA with input of cell metabolism genes in trained immunity (Fig.4B) indicate glycolysis are inhibited in the early stage of MERS infection, while recovered in late stage in endothelial cells; Nineteen molecules associate with Glycolysis I show opposite DE at 12 and 48 HPI; B Comparisons of the response of selected cell metabolism genes between EC in MERS (transcription level) and Caco-2 cells in SARS-COV-2 (translation and protein level, PMID: 32408336) during infections.

Supplementary Figure. 2

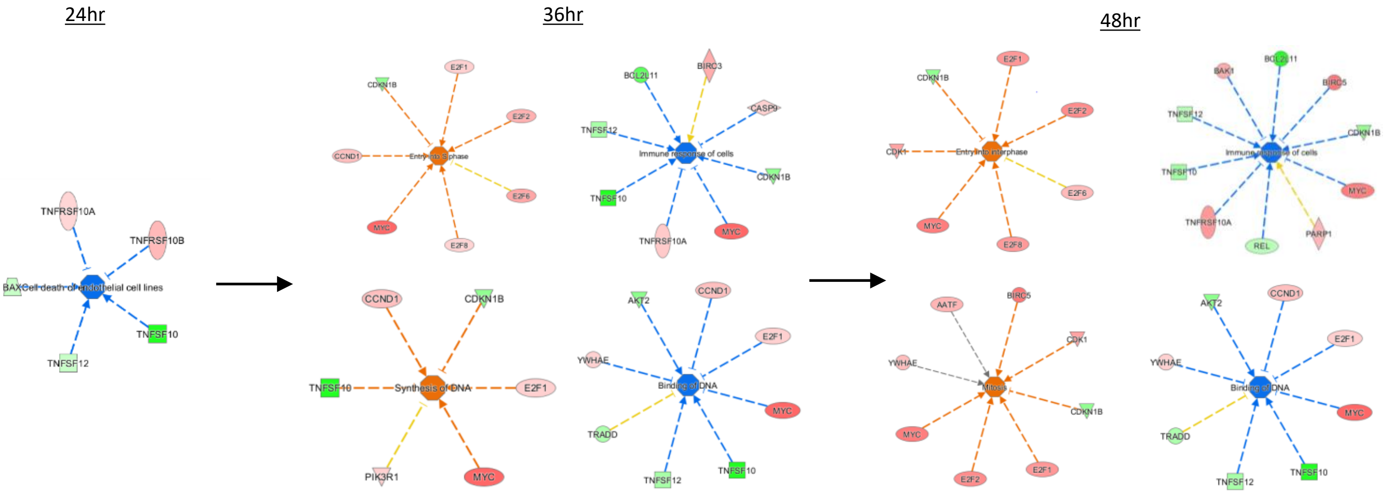

Diseases or Functions Annotation	Activation z-score		
	24hr	36hr	48hr
Cell death of endothelial cell lines	-2.236		
Synthesis of DNA		2.093	
Entry into S phase		2.106	
Binding of DNA		-2.076	-2.041
Immune response of cells		-2.067	-2.09
Entry into interphase			2.059
Mitosis			2.389

Supplementary Figure 2. (As a comparison) Disease and Function prediction generated from IPA based on the gene change of Apoptosis. Z- scores are included in the table. No significance were found in 12h PI.

Supplementary Figure. 3

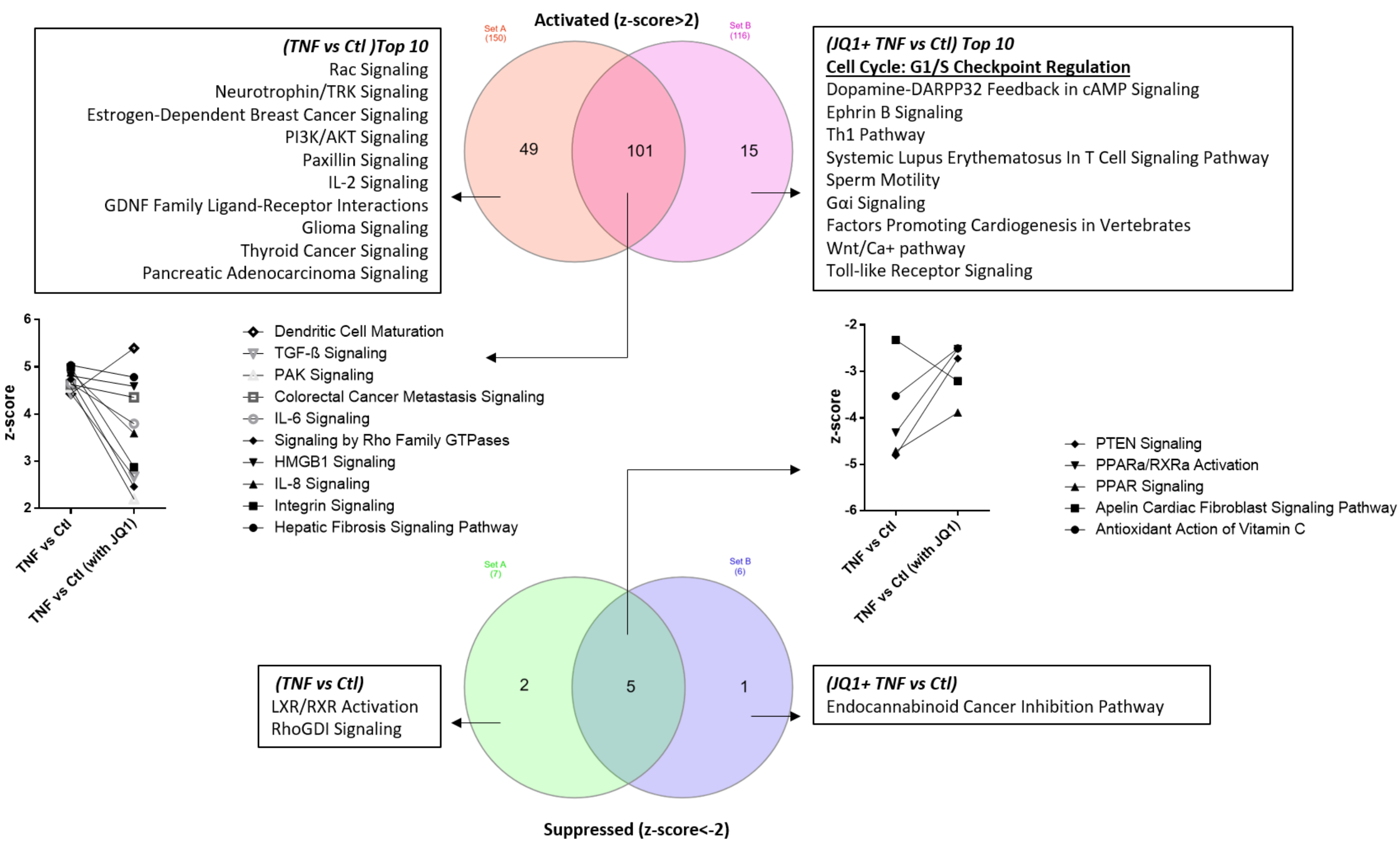

Supplementary Figure 3. G1/S checkpoint regulation pathway is activated by JQ1 application in the condition of TNF-alpha exposure. Results are obtained from IPA with the input of comparisons from GSE53999.
